# Health risk factors associated with meat, fruit and vegetable consumption in cohort studies: A comprehensive meta-analysis

**DOI:** 10.1371/journal.pone.0183787

**Published:** 2017-08-29

**Authors:** Giuseppe Grosso, Agnieszka Micek, Justyna Godos, Andrzej Pajak, Salvatore Sciacca, Fabio Galvano, Paolo Boffetta

**Affiliations:** 1 Integrated Cancer Registry of Catania-Messina-Siracusa-Enna, Azienda Ospedaliera Policlinico-Universitaria “Vittorio Emanuele”, Catania, Italy; 2 The Need for Nutrition Education/Innovation Programme (NNEdPro), University of Cambridge, Cambridge, United Kingdom; 3 Department of Epidemiology and Population Studies, Jagiellonian University Medical College, Krakow, Poland; 4 Department of Biomedical and Biotechnological Sciences, University of Catania, Catania, Italy; 5 Tisch Cancer Institute, Icahn School of Medicine at Mount Sinai, New York, NY, United States of America; University of South Alabama Mitchell Cancer Institute, UNITED STATES

## Abstract

The aim of this study was to perform a meta-analysis to test the association between red, processed, and total meat, as well as fruit and vegetable consumption, and selected health risk factors, including body weight status, smoking habit, physical activity level, level of education, and alcohol drinking in cohort studies on non-communicable disease. A systematic search of electronic databases was performed to identify relevant articles published up to March 2017. In a two-stage approach, frequency-weighted linear regression coefficients were first calculated for each variable, and then combined across studies through meta-regression. Ninety-eight studies including 20 on red meat, 6 on processed meat, 12 on total meat, 37 on fruit and vegetable combined, 21 on fruit and 24 on vegetable consumption were analyzed. Intake of red meat was positively associated with BMI, percentage of overweight and obese, low physical activity, and current and ever smoking and inversely associated with percentage of non-smokers and high physically active individuals. Similar associations were found for red meat were found, although based on fewer data. Intake of fruits and vegetables was positively associated with prevalence of non-smokers, high education and high physical activity, and similar results were found when examining fruit and vegetable consumption separately. Stratification by geographical area revealed that some associations were stronger in US rather than European or Asian cohorts. In conclusions, the distribution of health risk factors associated with high meat and fruit/vegetable consumption may differ from those of low-consumers. Some of these differences may mediate, confound, or modify the relation between diet and non-communicable disease risk.

## Introduction

Consumption of meat, fruit and vegetable has been the focus of epidemiologic research for its potential association with non-communicable diseases, including cancer risk. Findings from meta-analyses of observational studies indicate that consumption of meat would increase the risk of several cancers [1]. Based on hypotheses supporting the biological plausibility of such association, the Diet and Cancer Report published by the World Cancer Research Fund and American Institute for Cancer Research in 2007 concluded that the positive association between red and processed meat and colorectal cancer was convincing [2]. More recently, the International Agency for Research on Cancer in Lyon, France, published a monograph based on about 800 epidemiological studies that investigated the consumption of meat in relation to cancer risk [3]. The working group observed a significant association between intake of processed meat (meat that has been preserved by smoking, curing, salting, or by adding chemical preservatives) and colorectal cancer risk in 12 out of the 18 prospective cohort studies that provided relevant data [3]. Fourteen cohort studies were meta-analyzed to test the potential association between intake of red meat (meat from animals that have a high proportion of red muscle fibers) and several cancers, despite findings across studies were not consistent [3]. Similar uncertainty has been found on the association between fruits and vegetable consumption and cancer risk [4]. Epidemiological studies have produced contrasting evidence, showing decreased risk of head and neck, esophageal and stomach cancer and a non-linear decreased risk of colorectal cancer and puzzling results on other cancer sites [5]. Also the findings from a pooled analysis of 14 cohort studies are not convincing, reporting that fruit and vegetable intake was not strongly associated with colon cancer risk but may be associated with distant colon cancers [6].

These results have put in question the role of meat in human nutrition [7, 8]. Despite the great amount of scientific progress on the topic, causal link between diet and cancer is complex to demonstrate due to the concomitant consumption of several foods and nutrients, a possible association between unhealthy dietary and lifestyle habits, and methodological limitations in controlling potential confounding and distinguishing between risk factors and mediating effect of variables of interest. Most of the studies included in the reviews mentioned above focused on individual dietary components and adjusted for confounding factors which may be associated with the outcome of interest. Nonetheless, only small efforts have been done to assess whether a cluster of health risk factors associated with the variable of exposure may exist. The role of variables, such as adiposity, smoking habits, alcohol consumption, level of physical activity and education, has been widely assessed in affecting cancer risk [9]. It has been also shown that such variables tend to cluster with dietary habits and need to be targeted in health promotion interventions on multiple behaviors [10]. However, their association with meat (red, processed, and total), fruit and vegetable consumption has been addressed in individual studies [11] and few meta-analyses provided comparative risk estimates for such individual risk factors [12, 13], but no study comprehensively tested the potential association between intake of these food items and health risk factors. Thus, the aim of the present study was to evaluate the association between meat, fruit and vegetable intake and selected health risk factors in prospective cohort studies.

## Materials and methods

The reporting of this work is compliant with PRISMA guidelines (S1 Table).

### Study selection and data extraction

A systemic search of MEDLINE and EMBASE was performed to identify all articles published up to March 2017 including the keywords ‘meat’, ‘fruit’, and ‘vegetable’ combined with ‘cancer’ ‘cardiovascular’, ‘heart disease’, ‘stroke’, ‘mortality’, ‘hypertension’, ‘diabetes’ in order to collect information on existing cohorts investigated for meat, fruit and vegetable consumption. The inclusion criteria for the underlying studies were as follows: (i) prospective (cohort) design; (ii) results on the association between meat, including red, processed or total meat, or fruit and/or vegetable and any of the aforementioned outcomes; (iii) inclusion of at least one of lifestyle/background (smoking status, physical activity level, alcohol consumption, education level) and biomedical [body mass index (BMI) and weight status] characteristics as confounding variables; (iv) results for at least 3 categories of the exposure variable(s), either expressed as grams per day of intake, or reported in a way that could be converted in grams per day (i.e., servings per day); (v) English language. A search of the references cited in all of the articles selected for review was also conducted. When data of interest on a cohort was available in more than one study, the studies (i) including more individuals, (ii) providing separate information by sex, (iii) providing more variables, and (iv) providing more categories of exposure were selected. Study quality was assessed by applying the STrengthening the Reporting of OBservational studies in Epidemiology (STROBE) checklist for cohort studies [14].

Data extracted for each category of exposure included (i) number of individuals (cases and non-cases); (ii) median/mean amount of red, processed, and total meat, fruit, vegetable, or fruit and vegetable combined; and (iii) background characteristics, including: mean/median body mass index (BMI); proportion of individuals with BMI >25 and BMI >30; current, former, ever and never smokers; high-school/vocational and college/university education; individuals with high and low physical activity; and mean/median intake of alcohol. The extraction was performed by two researchers (GG and JG) and any disagreements were solved after discussion.

### Statistical analysis

When the range of consumption of the investigate exposures was given in the study, the midpoint of the interval was considered for the analysis. For open-ended categories of consumption, we assumed the same width as the adjacent one. One serving was approximated to 100 g when not otherwise specified. When food/serving was expressed as ratio per 1,000 kcal diet, the amount was doubled (approximately referring to a diet of 2,000 kcal). Background characteristics were graphically plotted by category of exposure to evaluate possible associations between dietary exposures and health risk factors.

The linearity of the associations between dietary exposures and health risk factors were tested using R^2^ and retained the association for which at least 50% of datasets had R^2^ >0.5. Moreover, diagnostic plots were generated to test the independence between the residuals and the fitted values. We found that in most of datasets (ranging from 50% to 100%, depending on the variable explored) the coefficient of determination R^2^ was rather high (i.e., >0.7), without a discernible pattern. However, a low percentage of datasets (i.e., <40%) fitted properly the model for the associations between red meat and low physical activity; processed meat and vegetable intake; and total meat and current smokers. We therefore did not consider further these associations.

Separate bivariate meta-analyses were performed for potential confounders to determine whether it was significantly associated with the exposures of interest. We used a two-stage approach meta-analysis [15]. First, for each exposure variable of interest within each study, ordinary least squares linear regression with frequency weights was used to estimate the coefficients for the intercepts and slopes of the dose-response associations between exposures and outcomes/confounders [16]. In the second step we synthesized the intercept and slope coefficients using bivariate meta-regression. Within each study, intercept and slope were often correlated and we obtained an overall regression model (rather than a single slope estimate). To synthesize vectors of coefficients we used generalized least squares, primarily because of the unequal variances of effects for studies of different sizes [16]. From the first step, we extracted the estimated model coefficients (intercepts and slopes) and the corresponding variance-covariance matrices of the sampling errors. We allowed for heterogeneity in the coefficients and allowed them to be correlated by using an unstructured variance-covariance matrix for the true outcomes. To further test for evidence of publication bias, subgroup analyses by sex and geographical area (US, Europe and Asia) were conducted. All analyses were performed with on R, software version 3.0.3 (Development Core Team, Vienna, Austria).

## Results

### Study characteristics

**Fig 1:**
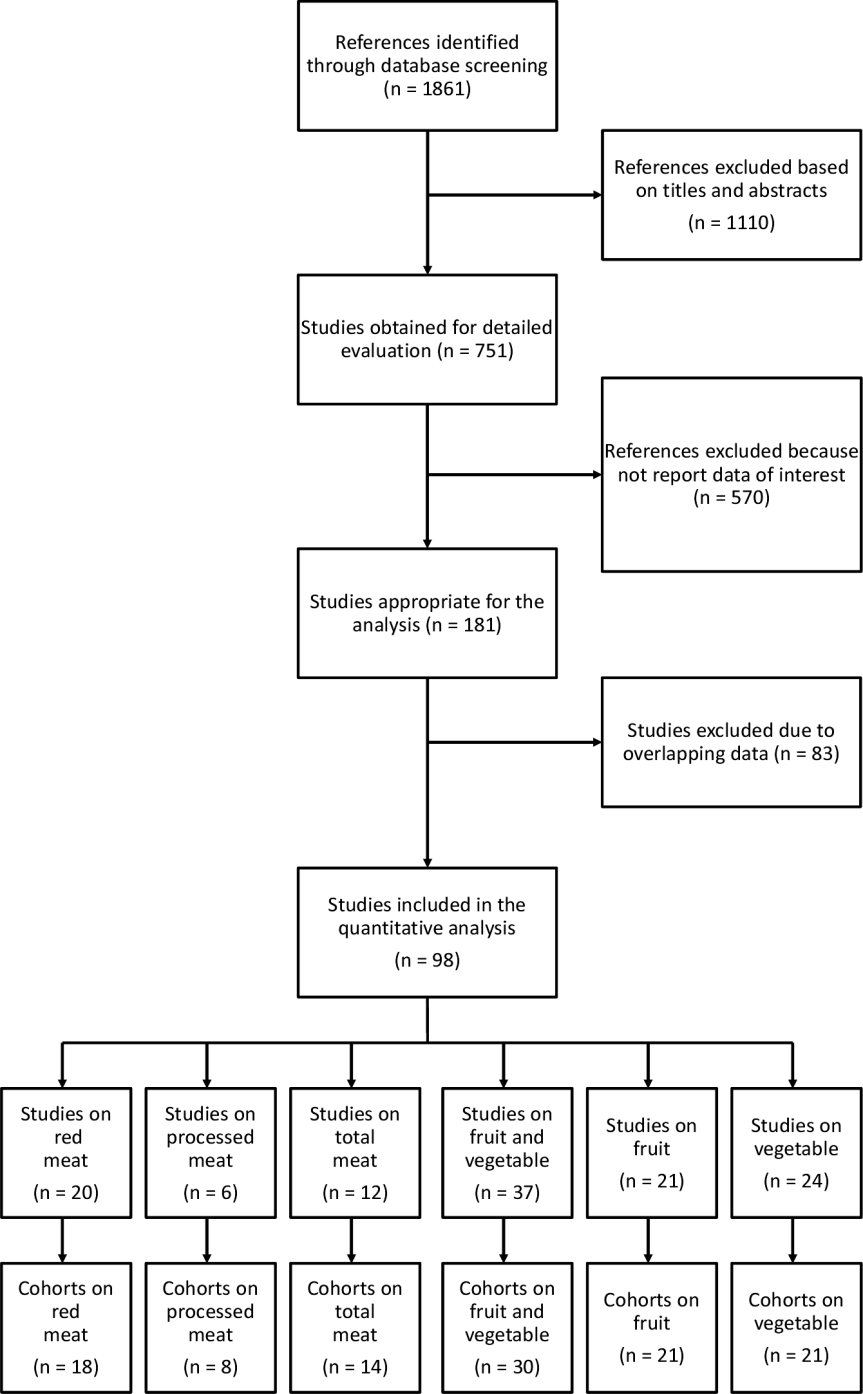
Screening and selection process of the study articles exploring the association between meat, fruit, vegetable consumption and cancer risk.

The search strategy resulted in 1,239 citations, 751 of which were potentially relevant and were retrieved as full-text articles (Fig 1). A total of 653 studies were excluded because they either did not report data of interest (n = 570) or were duplicates of more complete reports from the same cohorts (n = 83).

Therefore, a total of 98 univocal references were included in this meta-analysis [17–114], divided as follow: 20 studies on red meat [17–36], 6 on processed meat [26, 37–41], 12 on total meat [42–53], 37 on combined fruit and vegetable [37, 54–89], 21 on fruit [73, 90–109], and 24 on vegetable consumption [73, 90–96, 99–114]. All studies included were fully compliant to the STROBE statement. The list and main characteristics of the cohorts included are listed in S2 and S3 Tables. There were 18 unique cohorts on red meat, 8 on processed meat, 14 on total meat, 30 on combined fruit and vegetable, 21 on fruit and 21 on vegetable consumption. Number of individuals included varied on the basis of the association investigated, accounting for up to one million and half in most of analyses on the relation between red meat and fruit+vegetable intake and BMI levels and smoking status, while reaching up to 2 millions individuals in the analyses on educational status.

### Variables associated with meat consumption

**Fig 2:**
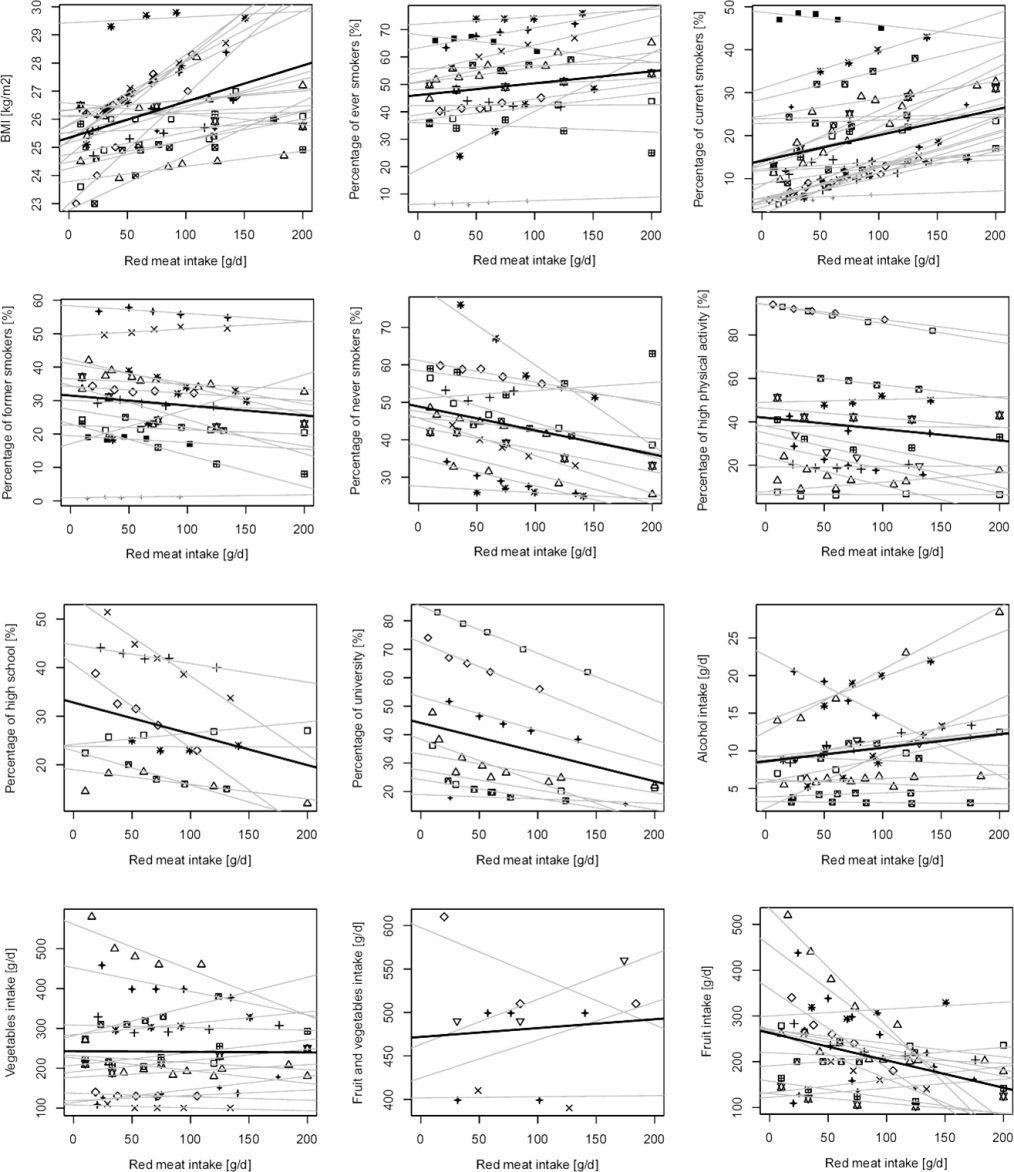
Scatter plot for associations between red meat consumption and baseline characteristics in prospective cohorts. Symbols represent different cohorts; light lines represent linear regression coefficients of individual studies; bold lines represent summary estimates average increase of each variable for increase of red meat intake.

The association between red meat consumption and the potential confounding factors is shown in Fig 2.

Low intake of red meat was associated with borderline BMI values for overweight [intercept coefficient 25.3; 95% confidence interval (CI): 24.7, 25.9] and with prevalence of overweight and obese individuals (Table 1).

**Table 1:** Summary associations between selected variables and red meat consumption.

Variables	No. of studies	No. of datasets	No. of cohorts	No. of individuals	Intercept (95% CI)	Slope per 100 g/d (95% CI)
BMI (mean/median)	13	20	14	1,650,663	25.34 (24.71, 25.97)	1.28 (0.74, 1.83)
BMI >30 (%)	2	2	2	52,441	14.32 (13.76, 14.88)	8.32 (7.18, 9.45)
BMI >25 (%)	3	4	3	133,099	43.34 (22.05, 64.62)	7.46 (6.23, 8.69)
Current smokers (%)	14	22	15	1,704,807	14.11 (9.27, 18.95)	5.96 (4.18, 7.75)
Former smokers (%)	8	14	8	1,230096	31.46 (23.97, 38.95)	-2.98 (-5.34, -0.62)
Ever smokers (%)	8	14	8	1,230096	46.05 (36.73, 55.37)	4.33 (1.34, 7.33)
Never smokers (%)	7	12	7	1,149438	48.86 (41.27, 56.44)	-6.39 (-9.29, -3.48)
High physical activity (%)	8	13	8	1,257227	41.82 (26.58, 57.06)	-5.22 (-8.41, -2.04)
Low physical activity (%)	3	5	3	519,118	15.22 (11.33, 19.12)	2.21 (0.32, 4.1)
Vocational/high school (%)	4	7	4	652,074	32.79 (22.67, 42.9)	-6.44 (-12.08, -0.8)
College/university (%)	5	8	5	1,177,778	44.09 (27.47, 60.7)	-10.26 (-14.16, -6.36)
Alcohol (g/d, mean/median)	10	14	11	1,409,369	8.61 (5.88, 11.34)	1.78 (-0.3, 3.87)
Fruit (g/d, mean/median)	9	14	10	1,310,820	262.59 (198.63, 326.56)	-59.84 (-106.23, -13.46)
Vegetable (g/d, mean/median)	9	14	10	1,310,820	242.59 (173.98, 311.19)	-1.62 (-25.72, 22.47)
Fruit+vegetable (g/d, mean/median)	2	4	4	195,985	471.75 (385.83, 557.66)	10.18 (-38.25, 58.61)

A100 gram per day increase in red meat consumption was associated with increased BMI [1.2 kg/m^3^; 95% confidence interval (CI): 0.7, 1.8] as well as increased proportion of overweight (7.4%, 95% CI: 6.2%, 8.6%) and obese individuals (8.3%, 95% CI: 7.1%, 9.4%) and current and ever smokers and decreased proportion of former and non-smokers (Table 1). Furthermore, there were associations with decreased prevalence of high physical activity. Prevalence of individuals with high-school/vocational and college/university education decreased by 6.4% and 10.2% for each 100 g/d increase of red meat, respectively (Table 1). Regarding other dietary variables, only fruit intake was significantly lower of about 60 g/d for each 100 g/d of red meat consumed, while no association was found with alcohol, vegetable, and combined fruit and vegetable consumption (Table 1). When analyses were stratified by sex, results did not change except for the associations with high physical activity in men and with fruit intake in women, which were no longer statistically significant (S4 Table); notably, the increase in BMI was stronger in women than in men (1.6 vs. 0.9 in women and men, respectively, for each 100 g/d of red meat). Associations with BMI and smoking status were consistent across regions, while the association with BMI was more evident in US than in European cohorts (1.8 vs. 0.5 increase in BMI each 100 g/d increased consumption of red meat, respectively) while no association was found in Asian cohorts (S5 Table). Moreover, the associations with physical activity were significant only among US cohorts (S5 Table), while the association with alcohol consumption was significant only in European cohorts (S5 Table). Finally, the associations with other dietary factors (i.e., fruit intake) were significant in the Asian and US cohorts but not in the European ones (S5 Table).

Fewer studies reported results on processed meat compared to red meat (S1 Fig). In general, characteristics of the variables of interest for low processed meat intake were similar to those identified for low red meat consumers (S6 Table). Results concerning BMI, smoking and education status were similar than those related to red meat; however, the associations were stronger than those observed for red meat, as each 100 g/d increase of processed meat was associated with increased BMI of 4.5 (95% CI: 1.6, 7.5), increased 25% of current smokers and decreased 31.4% and 17.1% of never smokers and college/university educated, respectively (S6 Table). Finally, increased intake of 100 mg/d of processed meat was associated with decreased intake of nearly 300 mg/d of fruit (S6 Table). Subgroup analyses revealed that increasing BMI values over increasing categories of processed meat intake were significant only for women, trends on educational status were significant only for men, while trends over smoking status were reported in both sexes (S7 Table). Subgroup analysis by geographical region provided further insights on potential differences between populations, as results were significant in both European and US cohorts, but range of variation of all the aforementioned variables was larger in the latter than in the former (S8 Table).

Studies reporting on the associations between total meat consumption and the variables of interest included a number of individuals smaller than that for red meat, but larger than that for processed meat (S2 Fig). Low total meat consumption was associated with reduced BMI, despite the association was weaker than for red and processed meat (23.7, 95% CI: 23.3, 24.2; S9 Table). Trends of BMI values across increasing categories of total meat intake resulted significant, but l smaller than those observed for specific type of meat (0.5 BMI increase and 7.6% increased prevalence of obese individuals for each 100 g/d total meat increased intake); no other significant associations were found (S9 Table). Subgroup analyses by sex (S10 Table) and geographical area (S11 Table) showed that previous findings on BMI were significant only among women in both Europe and US, while other associations, such as increasing prevalence of smokers (ever) and decreasing of never smokers, were found. Regarding Asian cohorts, increased intake of total meat was significantly associated with decreasing prevalence of obese individuals, increasing prevalence of individuals with high-school/vocational education, lower intake of alcohol and higher intake of fruit (S11 Table).

### Variables associated with fruit and vegetable consumption

**Fig 3:**
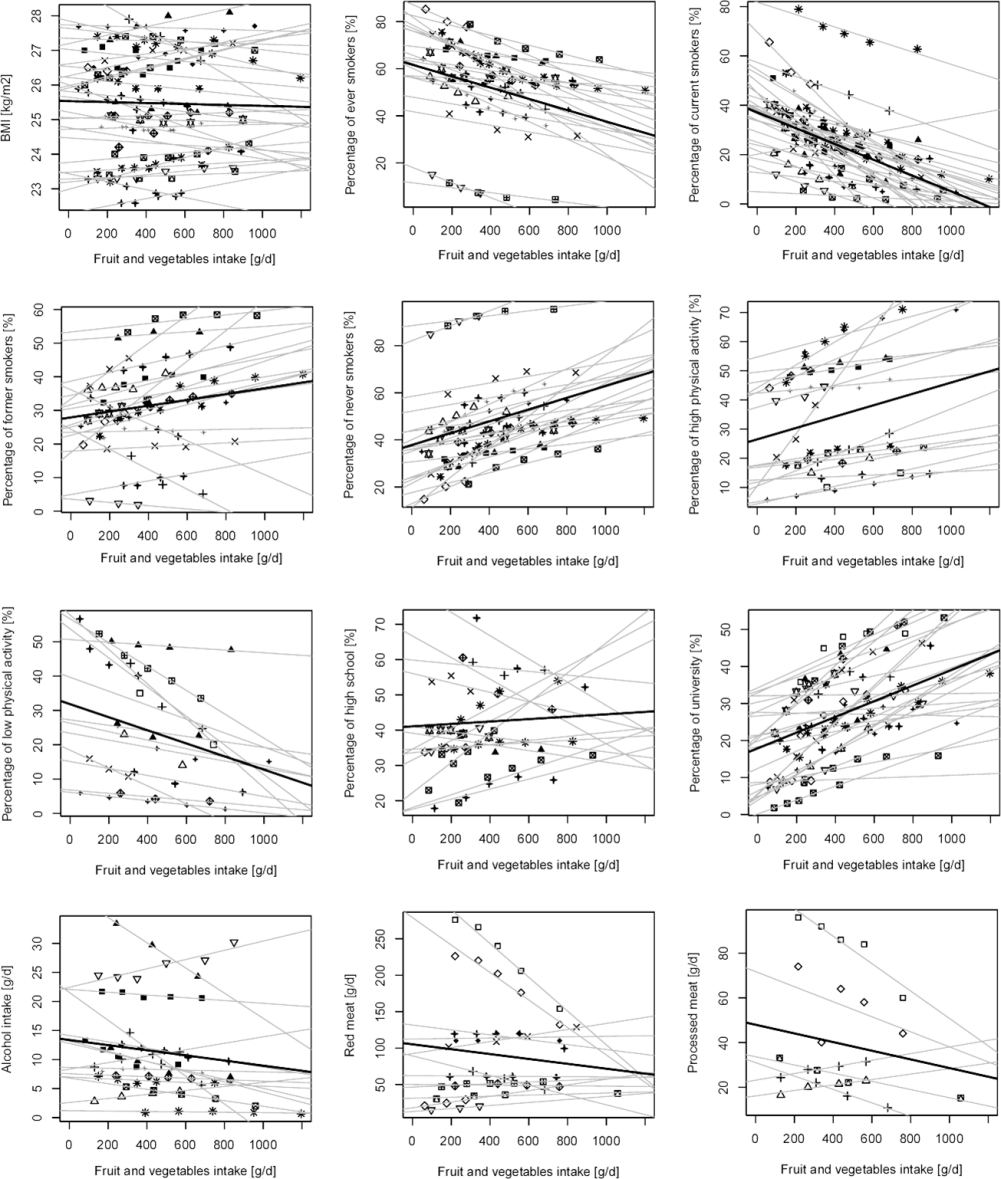
Scatter plot for associations between fruit and vegetable consumption and baseline characteristics in prospective cohorts. Symbols represent different cohorts; light lines represent linear regression coefficients of individual studies; bold lines represent summary estimates average increase of each variable for increase of fruit and vegetable intake.

The relations between fruit and vegetable consumption and the variables of interest are shown in Fig 3.

Low fruit and vegetable consumption was associated with BMI values as well as prevalence of overweight and obese similar to those observed for low red meat consumers, with no association with increased intakes of fruit and vegetable (Table 2). Pooled prevalence of ever (current + former) smokers in low fruit and vegetable consumers was slightly higher than in low meat consumers (roughly 60%) while that of non-smokers was lower (Table 2).

**Table 2:** Summary associations between selected variables and fruit+vegetable consumption.

Variables	No. of studies	No. of datasets	No. of cohorts	No. of individuals	Intercept (95% CI)	Slope per 100 g/d (95% CI)
BMI (mean/median)	23	29	25	1,618,453	25.53 (24.91, 26.15)	-0.01 (-0.05, 0.03)
BMI >30 (%)	6	6	6	289,158	18.47 (13.24, 23.7)	0.05 (-0.32, 0.43)
BMI >25 (%)	7	9	7	381,247	38.63 (25.21, 52.05)	-0.15 (-0.55, 0.26)
Current smokers (%)	23	30	26	1,809,001	37.06 (31.19, 42.93)	-3.19 (-3.92, -2.47)
Former smokers (%)	14	18	15	1,376,136	27.87 (21.89, 33.86)	0.87 (0.03, 1.71)
Ever smokers (%)	15	20	16	1,454,027	61.53 (52.77, 70.28)	-2.41 (-3.01, -1.81)
Never smokers (%)	15	20	16	1,454,027	37.76 (28.82, 46.7)	2.51 (1.9, 3.11)
High physical activity (%)	11	16	11	1,126,670	26.45 (17.71, 35.2)	1.95 (0.92, 2.97)
Low physical activity (%)	10	12	10	683,590	31.78 (20.92, 42.64)	-1.91 (-2.8, -1.01)
Vocational/high school (%)	10	13	11	801,769	41.05 (30.69, 51.42)	0.34 (-0.89, 1.57)
College/university (%)	18	24	19	2,229,993	17.87 (13.38, 22.36)	2.12 (1.52, 2.72)
Alcohol (g/d, mean/median)	10	14	12	1,242,020	13.35 (8.11, 18.59)	-0.44 (-0.92, 0.04)
Red meat (g/d, mean/median)	8	12	10	1,433,645	105.03 (47.27, 162.8)	-3.35 (-8.33, 1.63)
Processed meat (g/d, mean/median)	4	6	5	1,136,697	47.92 (18.37, 77.47)	-1.94 (-4.27, 0.4)

However, increased intake of 100 g/d of fruit and vegetable was associated with significant decreased prevalence of current (-3.1%; 95% CI: -3.9, -2.4) and ever smokers (-2.4; 95% CI: -3.0, -1.8), and increased prevalence of never smokers (2.5%; 95% CI: 1.9, 3.1). Another variable significantly associated with higher consumption of fruit and vegetable was prevalence of high (positively) and low (negatively) physically active individuals, which was roughly varying of +/- 2%, respectively, for each 100 g/d increased intake of fruit and vegetable. College/university education prevalence was also significantly associated with fruit and vegetable consumption (roughly increased of 2% each 100 g/d) while no association was found with alcohol or meat intake (Table 2). When analyses for fruit and vegetable intake were stratified by sex (S12 Table) and geographical region (S13 Table), all the associations remained significant for all subgroups examined. In addition, in Asian cohorts increasing intake of fruit and vegetable was also associated with increasing intake of alcohol (increase of 0.8 g/d of alcohol for 100 g/d increased intake of fruit and vegetable).

Similar pattern of associations were found when analyzing separately fruit (S3 Fig) and vegetable (S4 Fig) intake. Specifically, significant trends over current/never smokers, low/high physically active, and college/university education for increasing intake of both fruit (S14 Table) and vegetable consumption (S15 Table) were found. In contrast, 100 g/d increased intake of either fruit or vegetable was associated with decreased intake of alcohol (-3.2 g/d and -1.2 g/d, respectively), and individually with decreased intake of red meat (-3.1 g/d) and processed meat (-2.1 g/d), respectively. Furthermore, increased vegetable intake of about 100 g/d was associated with about 1% increased prevalence of overweight and obese individuals (S15 Table). The subgroup analyses revealed some slight differences in the associations, such as increased intake of fruit and vegetable consumption associated with education only among men and women, respectively, while both fruit and vegetable consumption was associated with physical activity only among women (S16 and S17 Tables). Small differences were found in the subgroup analysis by geographical region, as no associations were retrieved for fruit consumption in Asian cohorts and no association with education status in any of the subgroup areas (S18 Table); the association between vegetable consumption and the variable of interest were similar to those retrieved in the main analysis (S19 Table).

## Discussion

In this study we quantified the association between red and processed meat, as well as fruit and vegetable consumption, and some background characteristics in cohorts studied that provided results on the association between diet and cancer risk. There was a clear pattern with several health risk factors occurring in individuals consuming more meat (with a linear relation), including high BMI, obesity and current smoking rates, as well as low education and low consumption of fruits and vegetables. The examination of the same factors in relation to fruit and vegetable intake showed similar associations in the opposite direction (with the exception of BMI, substantially unrelated with the exposure), reinforcing the hypothesis that healthy and unhealthy dietary choices may cluster with health risk factors [115]. Moreover, while physical activity, smoking, and educational status may mediate the effects of meat, fruit and vegetable consumption toward health outcomes, body weight seems to be restricted to mediate the effects of the former. This was the first attempt to assess consistency of the underlying pattern of factors across cohorts exploring the association between meat, fruit and vegetable consumption and cancer risk. The present findings could explain the heterogeneity across results from cohort studies included in previous meta-analyses. It would be important to test the effect of such variables as moderators in diet-cancer associations, but this is not feasible based on the published results of these studies.

The covariates explored in this study in relation to red and processed meat intake have been long studied as potential risk factors for cancer. Increased risk of several cancers has been associated with higher BMI levels, including esophageal adenocarcinoma, thyroid, colon, and renal cancers, as well as endometrial, gallbladder, pancreas, and post-menopausal breast cancer in women [116]. Moreover, weight gain itself may increase the risk of some cancers, such as renal and postmenopausal breast, ovarian, and endometrial cancers [117]. Mechanisms of action linking body fat to cancer are not fully understood, but the main hypotheses regard alteration of body metabolism and hormonal systems as well as potential oxidative stress (initiated by hyperglycemia) that may play a role in tumorogenesis [118]. Meat intake can be associated to obesity due to its richness in fats or included in dietary patterns rich in processed foods, fries, and refined carbohydrates (especially processed meat). According to the results of the present study, BMI could represent one of the main confounders to explain the increased cancer risk associated with meat consumption. Noteworthy, data on BMI or obesity prevalence used for the present meta-analysis refer to baseline characteristics, and the possible effect of increase in BMI during the following of these longitudinal studies should be taken into account.

Tobacco smoking has been related to the majority of cancers [119]. Despite there is no direct biological relation between smoking and meat consumption, we found a strong and linear relation between these variables. It has been previously demonstrated that cigarette smoking was associated with unhealthy patterns of dietary factors, including higher intake of total energy, total fat, saturated fat, cholesterol and alcohol, and lower intake of polyunsaturated fat, fiber, vitamin C, vitamin E, and beta-carotene [120]. Interestingly, opposite associations were found in relation to fruit and vegetable consumption, with increasing intake associated with lower prevalence of smokers. Smoking habit can be part of a general unhealthy lifestyle that would also affect dietary habits and food choices, including excess of meat intake. Another finding reported in this meta-analysis, namely the association between meat consumption and lower educational status, could support the aforementioned hypothesis. Education level has been associated to cancer risk [121]. Lower educated individuals may be less aware of health issues related to certain behaviors including smoking habits and poor diet quality. In addition, lower education is a proxy of lower socio-economic status, which is also associated with poor health due to lack of resources and economic constraints. Among other possible confounders that have been associated to cancer risk and unhealthy behaviors [122], alcohol resulted only partially related with meat or fruit and vegetable consumption.

Another important confounding factor/effect modifier may be related to cultural and geographical aspects, as suggested by the stronger association between meat consumption and colorectal cancer risk in US than European studies [123]. Interestingly, differences of cancer risk between US and non-US cohorts have been recently shown also in relation to other foods, for instance eggs [124]. We tested this hypothesis by stratifying the analyses by sex and geographical area of the cohorts. As a result, certain differences were found, for instance red meat intake associated with physical activity and intake of fruit only in US cohorts (despite some other observations relied only on a limited number of cohorts); moreover, associations for weight, educational, and smoking status were stronger in US than in non-US cohorts.

The present study had several strengths, including the large number of people investigated, the large number of cohort form multiple countries with lifestyle and genetic heterogeneity, the high number of subgroup analyses, and consistency of results across different exposures and sensitivity analysis should assure the validity of our findings. Nevertheless, results should be considered in light of some limitations. First, the observational nature of the studies included does not allow defining causal relationships, rather only associations. Second, most of the studies did not specified whether “red meat” included “processed red meat” while only a minority provided this information as well as separate figures. Thus, we cannot exclude a certain degree of overlapping classification of type of exposure and we could only rely on authors’ description in the methodology for their definition of exposure, which in most of cases was exhaustive and should assure a very low rate of misclassification of exposure in our study. Moreover, most of results were similar between "red meat" and "processed meat", suggesting good reliability of our findings. Regarding the quantitative issue, most of data were retrieved from food frequency questionnaires, which is a better tool for ranking participants than estimating true amount of food consumption. Moreover, we estimated the exposure to meat or fruit/vegetable in several studies reporting only serving or other measures (i.e., cups equivalents). However, by using consistent conversions across studies, we should have minimized such issue. Third, a number of potentially relevant cohorts did not report sufficient information to be included in this meta-analysis. However, trends of associations of variables included in our models were quite consistent across studies, suggesting that there is no reason to suspect that results would change by considering also the missing cohorts. Finally, besides the well-known confounding factors examined in this study, residual confounders have not been taken into account in previous analyses or not uniformly comparable. Moreover, categorization of physical activity level was not uniform across studies (i.e., high physical activity may have referred to a certain amount of metabolic equivalents or a certain amount of leisure time physical activity or walking for at least a certain amount of time per week), thus results on this variable should be considered with caution. Regarding potential unknown factors, environmental contaminants may further alter the relation between raw/cooked meat and cancer risk [125]. Finally, the role of susceptibility to modifying genes involved in the metabolism of dietary carcinogens or anti-carcinogens following meat, fruit and vegetable consumption is still under consideration [126–128].

In conclusions, the results of this meta-analysis supported by the available data are largely consistent with a potential clustering of health risk factors that may confound the associations between food and cancer, in particular meat, fruit and vegetable. Due to practical and ethical considerations, conducting long-term controlled randomized trial on meat consumption and health outcomes is not possible. The main issue when considering results from observational studies regards the presence of several covariates that may be related to both the exposure and the outcome. The effects of individual foods or nutrients retrieved in observational studies cannot be studied in isolation. Adjusting for confounders may be incomplete and, in some cases, even not sufficient to limit their effect on the final risk estimate. A better understanding of the risk in population subgroups (i.e., smokers *vs*. non-smokers, normal weight *vs*. obese individuals, etc.) as well as the variation of the risk estimates by category of consumers of other foods (i.e., fruit and vegetable) or nutrients (fibre, antioxidants) would better elucidate and distinguish between risk factors and mere associations.

## Supporting information

S1 Table PRISMA checklist. (DOC)

S2 Table General information of the studies included for evaluation of variables associated with red, processed and total meat consumption. (DOCX)

S3 Table General information of the studies included for evaluation of variables associated with fruit and vegetable consumption. (DOCX)

S4 Table Summary associations between selected variables and red meat consumption, by sexes. NA, not applicable.
(DOCX)

S5 Table Summary associations between selected variables and red meat consumption, by geographical region. NA, not applicable.
(DOCX)

S6 Table Summary associations between selected variables and processed meat consumption. (DOCX)

S7 Table Summary associations between selected variables and processed meat consumption, by sexes. (DOCX)

S8 Table Summary associations between selected variables and processed meat consumption, by geographical region. NA, not applicable. (DOCX)

S9 Table Summary associations between selected variables and total meat consumption. (DOCX)

S10 Table Summary associations between selected variables and total meat consumption, by sexes. (DOCX)

S11 Table Summary associations between selected variables and total meat consumption, by geographical region. NA, not applicable.
(DOCX)

S12 Table Summary associations between selected variables and fruit+vegetable consumption, by sexes. (DOCX)

S13 Table Summary associations between selected variables and fruit+vegetable consumption, by geographical region. NA, not applicable.
(DOCX)

S14 Table Summary associations between selected variables and fruit consumption. (DOCX)

S15 Table Summary associations between selected variables and fruit consumption, by sexes. NA, not applicable.
(DOCX)

S16 Table Summary associations between selected variables and vegetable consumption. (DOCX)

S17 Table Summary associations between selected variables and vegetable consumption, by sexes. NA, not applicable.
(DOCX)

S18 Table Summary associations between selected variables and fruit consumption, by geographical region. NA, not applicable.
(DOCX)

S19 Table Summary associations between selected variables and vegetable consumption, by geographical region. (DOCX)

S1 Fig Scatter plot for associations between processed meat consumption and baseline characteristics in prospective cohorts. Symbols represent different cohorts; light lines represent linear regression coefficients of individual studies; bold lines represent summary estimates average increase of each variable for increase of red meat intake.
(TIF)

S2 Fig Scatter plot for associations between total meat consumption and baseline characteristics in prospective cohorts. Symbols represent different cohorts; light lines represent linear regression coefficients of individual studies; bold lines represent summary estimates average increase of each variable for increase of red meat intake.
(TIF)

S3 Fig Scatter plot for associations between fruit consumption and baseline characteristics in prospective cohorts. Symbols represent different cohorts; light lines represent linear regression coefficients of individual studies; bold lines represent summary estimates average increase of each variable for increase of fruit and vegetable intake.
(TIF)

S4 Fig Scatter plot for associations between vegetable consumption and baseline characteristics in prospective cohorts. Symbols represent different cohorts; light lines represent linear regression coefficients of individual studies; bold lines represent summary estimates average increase of each variable for increase of fruit and vegetable intake.
(TIF)
