# Supplementary material for: Health risk factors associated with meat, fruit and vegetable consumption in cohort studies: A comprehensive meta-analysis
Source: PLoS One. 2017 Aug 29;12(8):e0183787. doi: 10.1371/journal.pone.0183787 (PMC5574618; doi:10.1371/journal.pone.0183787)
Supplement: S2 Table — (DOCX) [file pone.0183787.s002.docx]

Supplementary Table 2. General information of the studies included for evaluation of variables associated with red, processed and total meat consumption.

| **Cohort name** | **No. individuals** | **Sex** | **Exposure** | **Categories** | **Unit** | **BMI** | **BMI >25** | **BMI >30** | **Smoking status** | **Educational status** | **Physical activity** | **Alcohol intake** | **Fruit/vegetable intake** | **Ref.** |
| --- | --- | --- | --- | --- | --- | --- | --- | --- | --- | --- | --- | --- | --- | --- |
| AHS | 23,080 | M | red meat | q1-q5 | g/d | NA | x | x | x | x | x | NA | NA | [1] |
| COSM | 37,035 | M | red meat | q1-q4 | g/d | x | NA | NA | x | x | NA | x | x | [2] |
| CPS II | 69,664 | M | red meat | q1-q5 | g/w | x | NA | NA | x | x | NA | NA | NA | [3] |
| CPS II | 78,946 | F | red meat | q1-q5 | g/w | x | NA | NA | x | x | NA | NA | NA | [3] |
| DDCH | 22,832 | M | red meat | q1-q4 | g/d | NA | NA | NA | x | x | x | x | NA | [4] |
| DDCH | 28,156 | F | red meat | q1-q4 | g/d | NA | NA | NA | x | x | x | x | NA | [4] |
| EPIC | 142,602 | M | red+proc | q1-q5 | g/d | x | NA | NA | x | x | x | x | x | [5] |
| EPIC | 335,825 | F | red+proc | q1-q5 | g/d | x | NA | NA | x | x | x | x | x | [5] |
| GCS | 42,403 | MF | red meat | q1-q5 | serv/w | x | NA | NA | x | NA | x | NA | x | [6] |
| HPFS | 37,698 | M | red meat | q1-q5 | serv/d | x | NA | NA | x | NA | NA | x | x | [7] |
| HPFS | 21,461 | M | red meat | q1,q3,q5 | serv/d | NA | NA | NA | NA | NA | NA | NA | x^a^ | [8] |
| JPHC I+II | 38,462 | M | red meat | q1-q5 | g/d | NA | x | NA | x | NA | NA | NA | NA | [9] |
| JPHC I+II | 42,196 | F | red meat | q1-q5 | g/d | NA | x | NA | x | NA | NA | NA | NA | [9] |
| MC | 99,431 | MF | red meat | q1,q3,q5 | g/d/1000 kcal | x | NA | NA | NA | NA | NA | x | NA | [10] |
| MRC NSHD | 517 | M | red meat | q1-q3 | g/d | x | NA | NA | NA | NA | NA | NA | NA | [11] |
| MRC NSHD | 634 | F | red meat | q1-q3 | g/d | x | NA | NA | NA | NA | NA | NA | NA | [11] |
| NHANES III | 8,239 | M | red meat | q1-q5 | serv/w | NA | NA | NA | x | NA | x | NA | NA | [12] |
| NHANES III | 9,372 | F | red meat | q1-q5 | serv/w | NA | NA | NA | x | NA | x | NA | NA | [12] |
| NHS I | 83,644 | F | red meat | q1-q5 | serv/d | x | NA | NA | x | NA | NA | x | x | [7] |
| NHS I | 47,256 | F | red meat | q1,q3,q5 | serv/d | x | NA | NA | x | NA | NA | x | x | [8] |
| NHS II | 90,651 | F | red meat | q1-q5 | serv/d | x | NA | NA | x | NA | NA | x | NA | [13] |
| NHS II | 52,623 | F | red meat | q1,q3,q5 | serv/d | x | NA | NA | x | NA | NA | x | x | [8] |
| NIH-AARP | 279,724 | M | red meat | q1-q5 | g/d/1000 kcal | x | NA | NA | x | x | x | x | x | [14] |
| NIH-AARP | 199,312 | F | red meat | q1-q5 | g/d/1000 kcal | x | NA | NA | x | x | x | x | x | [14] |
| PLCO | 29,361 | M | red meat | q1-q5 | g/d | NA | x | x | x | NA | x | NA | x | [15] |
| PLCO | 48,229 | M | red meat | q1-q5 | g/d/1000 kcal | x | NA | NA | x | x | NA | NA | x | [16] |
| PLCO | 51,350 | F | red meat | q1-q5 | g/d/1000 kcal | x | NA | NA | x | x | NA | NA | x | [16] |
| PLCO | 17,072 | MF | red meat | q1-q4 | g/d/1000 kcal | x | NA | NA | x | x | x | x | NA | [17] |
| PREDIMED | 717 | MF | red meat | q1-q4 | g/d | x | NA | NA | x | NA | NA | x | x | [18] |
| SMC | 34,670 | F | red meat | q1-q5 | g/d | x | NA | NA | x | x | NA | x | x | [19] |
| SMC+COSM | 74,645 | MF | red meat | q1-q5 | g/d | x | NA | NA | x | x | NA | NA | x | [20] |
| COSM | 37,035 | M | proc meat | q1-q4 | g/d | x | NA | NA | x | x | NA | x | x | [2] |
| E3N | 44,616 | F | proc meat | q1-q4 | serv/w | x | NA | NA | x | x | NA | x | x | [21] |
| HPFS | 28,410 | M | proc meat | q1,q3,q5 | g/d | x | NA | NA | x | NA | NA | x | x | [22] |
| NHS I | 53,131 | F | proc meat | q1,q3,q5 | g/d | x | NA | NA | x | NA | NA | x | x | [22] |
| NHS II | 57,019 | F | proc meat | q1,q3,q5 | g/d | x | NA | NA | x | NA | NA | x | x | [22] |
| NIH-AARP | 322,347 | M | proc meat | q1-q5 | OE/1000 kcal | NA | NA | NA | NA | x | NA | NA | x | [23] |
| NIH-AARP | 223,423 | F | proc meat | q1-q5 | OE/1000 kcal | NA | NA | NA | NA | x | NA | NA | x | [23] |
| SHFS | 2,001 | MF | proc meat | q1-q4 | g/d/1000 kcal | x | NA | NA | x | NA | NA | NA | x | [24] |
| SMC | 61,433 | F | proc meat | q1-q3 | serv/w | x | NA | NA | x | x | NA | x | x | [25] |
| ATBC | 25,943 | M | total meat | q1-q5 | g/d | x | NA | NA | NA | NA | x | x | x | [26] |
| BCDDP | 45,496 | F | total meat | q1-q5 | g/d/1000 kcal | x | NA | NA | x | x | NA | x | x | [27] |
| CLUE II | 3,892 | M | total meat | q1-q3 | g/d | x | NA | NA | x | NA | NA | x | NA | [28] |
| JACC | 20,466 | M | total meat | q1-q5 | g/d | x | NA | NA | x | x | x | x | x | [29] |
| JACC | 31,218 | F | total meat | q1-q5 | g/d | x | NA | NA | x | x | x | x | x | [29] |
| JPHC I+II | 27,425 | M | total meat | q1-q4 | g/d | x | NA | NA | x | NA | NA | NA | NA | [30] |
| JPHC I+II | 36,424 | F | total meat | q1-q4 | g/d | x | NA | NA | x | NA | NA | NA | NA | [30] |
| MCS | 41,835 | MF | total meat | q1-q4 | g/d | NA | x | x | x | x | x | NA | NA | [31] |
| NCS | 4,998 | M | total meat | q1-q3 | serv/w | x | NA | NA | x | x | NA | x | x | [32] |
| NCS | 5,714 | F | total meat | q1-q3 | serv/w | x | NA | NA | x | x | NA | x | x | [32] |
| NHS I | 88,808 | F | total meat | q1-q5 | g/d | x | NA | NA | x | NA | NA | x | NA | [33] |
| NIH-AARP | 187,265 | M | total meat | q1-q5 | g/d/1000 kcal | NA | x | x | x | x | x | x | NA | [34] |
| NIH-AARP | 135,581 | F | total meat | q1-q5 | g/d/1000 kcal | NA | x | x | x | x | x | x | NA | [34] |
| SMC+COSM | 82,002 | MF | total meat | q1-q4 | serv/w | x | NA | NA | x | x | NA | x | NA | [35] |
| SUN | 18,527 | MF | total meat | q1-q3 | serv/w | x | NA | NA | x | NA | NA | x | x | [36] |
| UK WCS | 33,725 | F | total meat | q1-q4 | g/d | x | NA | NA | x | NA | NA | NA | NA | [37] |

References

1. Koutros S, Cross AJ, Sandler DP, Hoppin JA, Ma X, Zheng T, Alavanja MC, Sinha R (2008) Meat and meat mutagens and risk of prostate cancer in the Agricultural Health Study. Cancer Epidemiol Biomarkers Prev 17 (1):80-87. doi:10.1158/1055-9965.EPI-07-0392

2. Kaluza J, Akesson A, Wolk A (2014) Processed and unprocessed red meat consumption and risk of heart failure: prospective study of men. Circ Heart Fail 7 (4):552-557. doi:10.1161/CIRCHEARTFAILURE.113.000921

3. Chao A, Thun MJ, Connell CJ, McCullough ML, Jacobs EJ, Flanders WD, Rodriguez C, Sinha R, Calle EE (2005) Meat consumption and risk of colorectal cancer. JAMA 293 (2):172-182. doi:10.1001/jama.293.2.172

4. Egeberg R, Olsen A, Christensen J, Halkjaer J, Jakobsen MU, Overvad K, Tjonneland A (2013) Associations between red meat and risks for colon and rectal cancer depend on the type of red meat consumed. J Nutr 143 (4):464-472. doi:10.3945/jn.112.168799

5. Linseisen J, Rohrmann S, Bueno-de-Mesquita B, Buchner FL, Boshuizen HC, Agudo A, Gram IT, Dahm CC, Overvad K, Egeberg R, Tjonneland A, Boeing H, Steffen A, Kaaks R, Lukanova A, Berrino F, Palli D, Panico S, Tumino R, Ardanaz E, Dorronsoro M, Huerta JM, Rodriguez L, Sanchez MJ, Rasmuson T, Hallmans G, Manjer J, Wirfalt E, Engeset D, Skeie G, Katsoulis M, Oikonomou E, Trichopoulou A, Peeters PH, Khaw KT, Wareham N, Allen N, Key T, Brennan P, Romieu I, Slimani N, Vergnaud AC, Xun WW, Vineis P, Riboli E (2011) Consumption of meat and fish and risk of lung cancer: results from the European Prospective Investigation into Cancer and Nutrition. Cancer Causes Control 22 (6):909-918. doi:10.1007/s10552-011-9764-1

6. Farvid MS, Malekshah AF, Pourshams A, Poustchi H, Sepanlou SG, Sharafkhah M, Khoshnia M, Farvid M, Abnet CC, Kamangar F, Dawsey SM, Brennan P, Pharoah PD, Boffetta P, Willett WC, Malekzadeh R (2017) Dietary Protein Sources and All-Cause and Cause-Specific Mortality: The Golestan Cohort Study in Iran. Am J Prev Med 52 (2):237-248. doi:10.1016/j.amepre.2016.10.041

7. Pan A, Sun Q, Bernstein AM, Schulze MB, Manson JE, Stampfer MJ, Willett WC, Hu FB (2012) Red meat consumption and mortality: results from 2 prospective cohort studies. Arch Intern Med 172 (7):555-563. doi:10.1001/archinternmed.2011.2287

8. Pan A, Sun Q, Bernstein AM, Schulze MB, Manson JE, Willett WC, Hu FB (2011) Red meat consumption and risk of type 2 diabetes: 3 cohorts of US adults and an updated meta-analysis. Am J Clin Nutr 94 (4):1088-1096. doi:10.3945/ajcn.111.018978

9. Takachi R, Tsubono Y, Baba K, Inoue M, Sasazuki S, Iwasaki M, Tsugane S, Japan Public Health Center-Based Prospective Study G (2011) Red meat intake may increase the risk of colon cancer in Japanese, a population with relatively low red meat consumption. Asia Pac J Clin Nutr 20 (4):603-612

10. Ollberding NJ, Wilkens LR, Henderson BE, Kolonel LN, Le Marchand L (2012) Meat consumption, heterocyclic amines and colorectal cancer risk: the Multiethnic Cohort Study. Int J Cancer 131 (7):E1125-1133. doi:10.1002/ijc.27546

11. Wagemakers JJ, Prynne CJ, Stephen AM, Wadsworth ME (2009) Consumption of red or processed meat does not predict risk factors for coronary heart disease; results from a cohort of British adults in 1989 and 1999. Eur J Clin Nutr 63 (3):303-311. doi:10.1038/sj.ejcn.1602954

12. Kappeler R, Eichholzer M, Rohrmann S (2013) Meat consumption and diet quality and mortality in NHANES III. Eur J Clin Nutr 67 (6):598-606. doi:10.1038/ejcn.2013.59

13. Cho E, Chen WY, Hunter DJ, Stampfer MJ, Colditz GA, Hankinson SE, Willett WC (2006) Red meat intake and risk of breast cancer among premenopausal women. Arch Intern Med 166 (20):2253-2259. doi:10.1001/archinte.166.20.2253

14. Cross AJ, Leitzmann MF, Gail MH, Hollenbeck AR, Schatzkin A, Sinha R (2007) A prospective study of red and processed meat intake in relation to cancer risk. PLoS Med 4 (12):e325. doi:10.1371/journal.pmed.0040325

15. Cross AJ, Peters U, Kirsh VA, Andriole GL, Reding D, Hayes RB, Sinha R (2005) A prospective study of meat and meat mutagens and prostate cancer risk. Cancer Res 65 (24):11779-11784. doi:10.1158/0008-5472.CAN-05-2191

16. Tasevska N, Cross AJ, Dodd KW, Ziegler RG, Caporaso NE, Sinha R (2011) No effect of meat, meat cooking preferences, meat mutagens or heme iron on lung cancer risk in the prostate, lung, colorectal and ovarian cancer screening trial. Int J Cancer 128 (2):402-411. doi:10.1002/ijc.25327

17. Ferrucci LM, Sinha R, Huang WY, Berndt SI, Katki HA, Schoen RE, Hayes RB, Cross AJ (2012) Meat consumption and the risk of incident distal colon and rectal adenoma. Br J Cancer 106 (3):608-616. doi:10.1038/bjc.2011.549

18. Babio N, Sorli M, Bullo M, Basora J, Ibarrola-Jurado N, Fernandez-Ballart J, Martinez-Gonzalez MA, Serra-Majem L, Gonzalez-Perez R, Salas-Salvado J, Nureta PI (2012) Association between red meat consumption and metabolic syndrome in a Mediterranean population at high cardiovascular risk: cross-sectional and 1-year follow-up assessment. Nutr Metab Cardiovasc Dis 22 (3):200-207. doi:10.1016/j.numecd.2010.06.011

19. Larsson SC, Virtamo J, Wolk A (2011) Red meat consumption and risk of stroke in Swedish women. Stroke 42 (2):324-329. doi:10.1161/STROKEAHA.110.596510

20. Bellavia A, Stilling F, Wolk A (2016) High red meat intake and all-cause cardiovascular and cancer mortality: is the risk modified by fruit and vegetable intake? Am J Clin Nutr 104 (4):1137-1143. doi:10.3945/ajcn.116.135335

21. Lajous M, Bijon A, Fagherazzi G, Rossignol E, Boutron-Ruault MC, Clavel-Chapelon F (2014) Processed and unprocessed red meat consumption and hypertension in women. Am J Clin Nutr 100 (3):948-952. doi:10.3945/ajcn.113.080598

22. Michaud DS, Holick CN, Batchelor TT, Giovannucci E, Hunter DJ (2009) Prospective study of meat intake and dietary nitrates, nitrites, and nitrosamines and risk of adult glioma. Am J Clin Nutr 90 (3):570-577. doi:10.3945/ajcn.2008.27199

23. Dubrow R, Darefsky AS, Park Y, Mayne ST, Moore SC, Kilfoy B, Cross AJ, Sinha R, Hollenbeck AR, Schatzkin A, Ward MH (2010) Dietary components related to N-nitroso compound formation: a prospective study of adult glioma. Cancer Epidemiol Biomarkers Prev 19 (7):1709-1722. doi:10.1158/1055-9965.EPI-10-0225

24. Fretts AM, Howard BV, McKnight B, Duncan GE, Beresford SA, Mete M, Eilat-Adar S, Zhang Y, Siscovick DS (2012) Associations of processed meat and unprocessed red meat intake with incident diabetes: the Strong Heart Family Study. Am J Clin Nutr 95 (3):752-758. doi:10.3945/ajcn.111.029942

25. Larsson SC, Bergkvist L, Wolk A (2006) Processed meat consumption, dietary nitrosamines and stomach cancer risk in a cohort of Swedish women. Int J Cancer 119 (4):915-919. doi:10.1002/ijc.21925

26. Mannisto S, Kontto J, Kataja-Tuomola M, Albanes D, Virtamo J (2010) High processed meat consumption is a risk factor of type 2 diabetes in the Alpha-Tocopherol, Beta-Carotene Cancer Prevention study. Br J Nutr 103 (12):1817-1822. doi:10.1017/S0007114510000073

27. Flood A, Velie EM, Chaterjee N, Subar AF, Thompson FE, Lacey JV, Jr., Schairer C, Troisi R, Schatzkin A (2002) Fruit and vegetable intakes and the risk of colorectal cancer in the Breast Cancer Detection Demonstration Project follow-up cohort. Am J Clin Nutr 75 (5):936-943

28. Rohrmann S, Platz EA, Kavanaugh CJ, Thuita L, Hoffman SC, Helzlsouer KJ (2007) Meat and dairy consumption and subsequent risk of prostate cancer in a US cohort study. Cancer Causes Control 18 (1):41-50

29. Nagao M, Iso H, Yamagishi K, Date C, Tamakoshi A (2012) Meat consumption in relation to mortality from cardiovascular disease among Japanese men and women. Eur J Clin Nutr 66 (6):687-693. doi:10.1038/ejcn.2012.6

30. Kurotani K, Nanri A, Goto A, Mizoue T, Noda M, Oba S, Kato M, Matsushita Y, Inoue M, Tsugane S, Japan Public Health Center-based Prospective Study G (2013) Red meat consumption is associated with the risk of type 2 diabetes in men but not in women: a Japan Public Health Center-based Prospective Study. Br J Nutr 110 (10):1910-1918. doi:10.1017/S0007114513001128

31. Sato Y, Nakaya N, Kuriyama S, Nishino Y, Tsubono Y, Tsuji I (2006) Meat consumption and risk of colorectal cancer in Japan: the Miyagi Cohort Study. Eur J Cancer Prev 15 (3):211-218. doi:10.1097/01.cej.0000197455.87356.05

32. Gilsing AM, Weijenberg MP, Goldbohm RA, Dagnelie PC, van den Brandt PA, Schouten LJ (2013) The Netherlands Cohort Study-Meat Investigation Cohort; a population-based cohort over-represented with vegetarians, pescetarians and low meat consumers. Nutr J 12:156. doi:10.1186/1475-2891-12-156

33. Michaud DS, Giovannucci E, Willett WC, Colditz GA, Fuchs CS (2003) Dietary meat, dairy products, fat, and cholesterol and pancreatic cancer risk in a prospective study. Am J Epidemiol 157 (12):1115-1125

34. Taunk P, Hecht E, Stolzenberg-Solomon R (2016) Are meat and heme iron intake associated with pancreatic cancer? Results from the NIH-AARP diet and health cohort. Int J Cancer 138 (9):2172-2189. doi:10.1002/ijc.29964

35. Larsson SC, Johansson JE, Andersson SO, Wolk A (2009) Meat intake and bladder cancer risk in a Swedish prospective cohort. Cancer Causes Control 20 (1):35-40. doi:10.1007/s10552-008-9214-x

36. Mari-Sanchis A, Gea A, Basterra-Gortari FJ, Martinez-Gonzalez MA, Beunza JJ, Bes-Rastrollo M (2016) Meat Consumption and Risk of Developing Type 2 Diabetes in the SUN Project: A Highly Educated Middle-Class Population. PLoS One 11 (7):e0157990. doi:10.1371/journal.pone.0157990

37. Taylor EF, Burley VJ, Greenwood DC, Cade JE (2007) Meat consumption and risk of breast cancer in the UK Women's Cohort Study. Br J Cancer 96 (7):1139-1146. doi:10.1038/sj.bjc.6603689
