# Supplementary material for: Health risk factors associated with meat, fruit and vegetable consumption in cohort studies: A comprehensive meta-analysis
Source: PLoS One. 2017 Aug 29;12(8):e0183787. doi: 10.1371/journal.pone.0183787 (PMC5574618; doi:10.1371/journal.pone.0183787)
Supplement: S3 Table — (DOCX) [file pone.0183787.s003.docx]

Supplementary Table 3. General information of the studies included for evaluation of variables associated fruit and vegetable consumption.

| **Cohort name** | **No. individuals** | **Sex** | **Exposure** | **Categories** | **Unit** | **BMI** | **BMI >25** | **BMI >30** | **Smoking status** | **Educational status** | **Physical activity** | **Alcohol intake** | **Meat intake** | **Ref.** |
| --- | --- | --- | --- | --- | --- | --- | --- | --- | --- | --- | --- | --- | --- | --- |
| 45 and Up Study | 150,969 | MF | FV | q1-q4 | serv/d | NA | x | x | x | x | x | NA | NA | [1] |
| ARIC | 11,940 | MF | FV | q1-q5 | serv/d | x | NA | NA | x | x | x | x | NA | [2] |
| ATBC | 27,111 | M | FV | q1-q5 | g/d | x | NA | NA | NA | x | NA | NA | NA | [3] |
| CARDIA | 1,571 | F | FV | q1-q3 | serv/d/2000kcal | x | NA | NA | x | x | x | NA | NA | [4] |
| CARDIA | 935 | M | FV | q1-q3 | serv/d/2000kcal | x | NA | NA | x | x | x | NA | NA | [4] |
| COSM | 45,338 | M | FV | q1-q4 | serv/d | NA | NA | NA | x | x | NA | x | x | [5] |
| DDCHS | 54,505 | MF | FV | q1-q5 | g/d | x | NA | NA | x | x | NA | NA | NA | [6] |
| EPIC | 478,545 | MF | FV | q1-q5 | g/d | x | NA | NA | x | x | x | x | NA | [7] |
| EPIC | 131,985 | M | FV | q1-q5 | g/d | x | NA | NA | x | NA | x | x | NA | [8] |
| EPIC | 320,771 | F | FV | q1-q5 | g/d | x | NA | NA | x | NA | x | x | NA | [8] |
| EPIC | 451,151 | MF | FV | q1-q4 | g/d | x | NA | NA | x | x | x | x | x | [9] |
| HAPIEE | 19,333 | MF | FV | q1-q4 | g/d | x | NA | x | x | x | x | x | NA | [10] |
| HPFS | 47,778 | M | FV | q1-q5 | serv/d | x | NA | NA | x | NA | NA | x | NA | [11] |
| HPFS | 42,135 | M | FV | q1-q5 | serv/d | x | NA | NA | x | NA | NA | x | x | [12] |
| HSE | 65,226 | MF | FV | q1-q5 | serv/d | x | x | x | x | x | x | x | NA | [13] |
| IWHS | 35,159 | F | FV | q1-q5 | serv/d | x | NA | NA | x | x | NA | NA | x | [14] |
| JPHC I+II | 21,269 | M | FV | q1-q4 | g/d | x | NA | NA | x | NA | x | NA | NA | [15] |
| JPHC I+II | 27,168 | F | FV | q1-q4 | g/d | x | NA | NA | x | NA | x | NA | NA | [15] |
| JPHC I+II | 35,909 | M | FV | q1-q4 | g/d | NA | NA | x | x | NA | NA | NA | NA | [16] |
| JPHC I+II | 41,982 | F | FV | q1-q4 | g/d | NA | NA | x | x | NA | NA | NA | NA | [16] |
| KIHD | 2,641 | M | FV | q1-q5 | g/d | x | NA | x | x | NA | NA | X | NA | [17] |
| MORGEN | 20,070 | MF | FV | q1-q4 | g/d | x | NA | NA | x | x | x | NA | NA | [18] |
| NHEFS | 8,608 | MF | FV | q1-q4 | serv/d | x | NA | NA | x | x | NA | NA | NA | [19] |
| NHS | 77,283 | F | FV | q1-q5 | serv/d | x | NA | NA | x | NA | NA | x | NA | [11] |
| NHS | 71,141 | F | FV | q1-q5 | serv/d | x | NA | NA | x | NA | NA | x | x | [12] |
| NIH-AARP | 281,288 | M | FV | q1-q5 | serv/d/1000 | x | NA | NA | x | x | NA | x | NA | [20] |
| NIH-AARP | 190,793 | F | FV | q1-q5 | serv/d/1000 | x | NA | NA | x | x | NA | x | NA | [20] |
| NIH-AARP | 322,347 | M | FV | q1-q5 | serv/d/1000 | NA | NA | NA | NA | x | NA | NA | x | [21] |
| NIH-AARP | 223,423 | F | FV | q1-q5 | serv/d/1000 | NA | NA | NA | NA | x | NA | NA | x | [21] |
| NIH-AARP | 174,636 | M | FV | q1,q3,q5 | serv/d | x | NA | NA | x | x | x | NA | NA | [22] |
| NIH-AARP | 118,167 | F | FV | q1,q3,q5 | serv/d | x | NA | NA | x | x | x | NA | NA | [22] |
| NIPPON DATA80 Study | 9,732 | MF | FV | q1-q4 | g/d/1000 kcal | x | NA | NA | x | NA | NA | NA | NA | [23] |
| Odyssey Cohort (including CLUE I, CLUE II) | 6,151 | MF | FV | q1-q5 | serv/d | NA | x | x | x | NA | NA | x | NA | [24] |
| ONHICS | 16,065 | M | FV | q1-q3 | g/d | NA | x | NA | x | x | x | NA | NA | [25] |
| ONHICS | 16,794 | F | FV | q1-q3 | g/d | NA | x | NA | x | x | x | NA | NA | [25] |
| PLCO | 57,774 | MF | FV | q1-q3 | serv/d/1000 | x | NA | NA | x | x | x | x | x | [26] |
| PRIME | 8,540 | M | FV | q1-q3 | serv/d | x | NA | NA | NA | NA | NA | NA | NA | [27] |
| PRIME | 8,087 | M | FV | q1-q3 | serv/d | NA | NA | NA | x | x | x | NA | NA | [28] |
| SMC | 36,664 | F | FV | q1-q4 | serv/d | NA | NA | NA | x | x | NA | x | x | [5] |
| SMC | 34,319 | F | FV | q1-q5 | serv/d | x | NA | NA | x | x | NA | x | NA | [29] |
| SMC+COSM | 74,971 | MF | FV | q1-q5 | serv/d | NA | x | NA | x | x | NA | x | x | [30] |
| SMCS | 15,119 | M | FV | q1-q6 | g/d | x | NA | NA | NA | x | x | x | x | [31] |
| SMHS | 55,474 | M | FV | q1-q4 | g/d | x | NA | NA | x | x | NA | NA | x | [32] |
| SMHS | 61,436 | M | FV | q1-q5 | g/d | x | NA | NA | x | x | NA | NA | NA | [33] |
| SUN | 8,594 | MF | FV | q1-q4 | serv/d | x | NA | NA | x | NA | NA | x | NA | [34] |
| SUVIMAX | 3,313 | M | FV | q1-q3 | g/d | x | x | NA | x | x | x | x | NA | [35] |
| SWHS | 67,211 | F | FV | q1-q4 | g/d | x | NA | NA | x | x | NA | NA | x | [32] |
| SWHS | 73,360 | F | FV | q1-q5 | g/d | x | NA | NA | x | x | NA | NA | NA | [33] |
| SWLH | 44,838 | F | FV | q1-q5 | g/d |  | x | x | x | x | NA | NA | NA | [36] |
| The Framingham Study | 832 | M | FV | q1-q5 | serv/d | x | NA | NA | NA | NA | NA | NA | NA | [37] |
| 45 and Up Study | 150,969 | MF | F | q1-q4 | serv/d |  | x | x | x | x | x | NA | NA | [1] |
| BCDDP | 41,320 | F | F | q1-q4 | serv/d | x | NA | NA | x | x | NA | x | x | [38] |
| BWHS | 51,928 | F | F | q1-q4 | serv/w | x | NA | NA | x | x | x | NA | NA | [39] |
| CGPS+CCHS | 83,256 | MF | F | q1-q4 | serv/d | x | NA | NA | x | NA | x | x | NA | [40] |
| CPS II | 37,563 | M | F | q1,q3,q5 | serv/d | x | NA | NA | x | x | NA | NA | x | [41] |
| CPS II | 42,330 | F | F | q1,q3,q5 | serv/d | x | NA | NA | x | x | NA | NA | x | [41] |
| EPIC | 486,799 | MF | F | q1-q5 | g/d | NA | x | x | x | x | x | NA | NA | [42] |
| HPFS | 47,325 | M | F | q1-q5 | serv/d | x | NA | NA | NA | NA | NA | x | NA | [43] |
| HPFS | 23,208 | M | F | q1,q3,q5 | serv/d | x | NA | NA | x | NA | NA | NA | NA | [44] |
| JACC | 59,485 | MF | F | q1-q4 | serv/w | x | NA | NA | x | x | x | x | NA | [45] |
| JPHC I+II | 47,279 | F | F | q1-q4 | g/d | x | NA | NA | x | NA | NA | NA | NA | [46] |
| LSS | 38,540 | MF | F | q1-q3 | serv/w | x | NA | NA | x | x | NA | NA | NA | [47] |
| NHIS | 20,004 | MF | F | q1-q4 | serv/d | NA | NA | NA | x | NA | NA | NA | NA | [48] |
| NHS | 34,557 | F | F | q1-q5 | serv/d | x | NA | NA | x | NA | x | x | NA | [49] |
| NHS | 45,357 | F | F | q1,q3,q5 | serv/d | x | NA | NA | x | NA | x | NA | NA | [44] |
| NHS II | 54,196 | F | F | q1,q3,q5 | serv/d | x | NA | NA | x | NA | NA | x | x | [50] |
| PLCO | 17,621 | MF | F | q1,q3,q5 | serv/d | x | NA | NA | x | NA | NA | NA | x | [51] |
| SWHS | 64,191 | F | F | q1-q5 | g/d | x | x | x | x | x | x | NA | NA | [52] |
| Takayama Study | 13,355 | M | F | q1-q4 | g/d | x | NA | NA | x | x | NA | x | NA | [53] |
| Takayama Study | 15,724 | F | F | q1-q4 | g/d | x | NA | NA | x | x | NA | x | NA | [53] |
| The Study of Men Born in 1913 | 730 | M | F | q1-q4 | serv/w | x | NA | NA | x | NA | NA | NA | NA | [54] |
| UK WCS | 30,458 | F | F | q1-q5 | g/d | x | NA | NA | x | x | NA | x | NA | [55] |
| WHS | 22,144 | F | F | q1,q3,q5 | serv/d | x | NA | NA | x | x | NA | x | x | [56] |
| WHS | 39,127 | F | F | q1-q5 | serv/d | x | NA | NA | x | NA | x | NA | NA | [57] |
| 45 and Up Study | 150,969 | MF | V | q1-q4 | serv/d | NA | x | x | x | x | x | NA | NA | [1] |
| BCDDP | 41,320 | F | V | q1-q4 | serv/d | x | NA | NA | x | x | NA | x | x | [38] |
| BWHS | 51,928 | F | V | q1-q4 | serv/w | x | NA | NA | x | x | x | NA | NA | [39] |
| CPS II | 37,563 | M | V | q1,q3,q5 | serv/d | x | NA | NA | x | x | NA | NA | x | [41] |
| CPS II | 42,330 | F | V | q1,q3,q5 | serv/d | x | NA | NA | x | x | NA | NA | x | [41] |
| EPIC | 486,799 | MF | V | q1-q5 | g/d | NA | x | x | x | x | x | NA | NA | [42] |
| EPICOR | 29,689 | F | V | q1-q4 | g/d | x | NA | NA | x | x | x | x | NA | [58] |
| HPFS | 47,325 | M | V | q1-q5 | serv/d | x | NA | NA | NA | NA | NA | x | NA | [43] |
| HPFS | 23,208 | M | V | q1,q3,q5 | serv/d | x | NA | NA | x | NA | NA | NA | NA | [44] |
| JACC | 59,485 | MF | V | q1-q4 | serv/w | x | NA | NA | x | x | x | x | NA | [45] |
| JPHC I | 20,366 | M | V | q1-q3 | g/d | x | NA | NA | x | NA | x | NA | NA | [59] |
| JPHC I | 21,858 | F | V | q1-q3 | g/d | x | NA | NA | x | NA | x | NA | NA | [59] |
| JPHC I+II | 43,475 | M | V | q1-q4 | g/d | NA | x | NA | x | NA | NA | NA | NA | [60] |
| JPHC I+II | 47,288 | F | V | q1-q4 | g/d | x | NA | NA | x | NA | NA | NA | NA | [46] |
| JPHC II | 24,407 | M | V | q1-q3 | g/d | x | NA | NA | x | NA | x | NA | NA | [59] |
| JPHC II | 26,708 | F | V | q1-q3 | g/d | x | NA | NA | x | NA | x | NA | NA | [59] |
| LSS | 38,540 | MF | V | q1-q3 | serv/d | x | NA | NA | x | x | NA | NA | NA | [47] |
| MC | 183,522 | MF | V | q1-q5 | g/d/1000 kcal | x | NA | NA | x | NA | NA | NA | x | [61] |
| NHIS | 20,004 | MF | V | q1-q4 | serv/d | NA | NA | NA | x | NA | NA | NA | NA | [48] |
| NHS | 34,467 | F | V | q1-q5 | serv/d | x | NA | NA | x | NA | x | x | NA | [49] |
| NHS | 45,357 | F | V | q1,q3,q5 | serv/d | x | NA | NA | x | NA | x | NA | NA | [44] |
| NHS II | 54,298 | F | V | q1,q3,q5 | serv/d | x | NA | NA | x | NA | NA | x | x | [50] |
| PLCO | 17,616 | MF | V | q1,q3,q5 | serv/d | x | NA | NA | x | NA | NA | NA | x | [51] |
| SWHS | 64,191 | F | V | q1-q5 | g/d | x | x | x | x | x | x | NA | NA | [52] |
| Takayama Study | 13,355 | M | V | q1-q4 | g/d | x | NA | NA | x | x | NA | x | NA | [53] |
| Takayama Study | 15,724 | F | V | q1-q4 | g/d | x | NA | NA | x | x | NA | x | NA | [53] |
| The Study of Men Born in 1913 | 729 | M | V | q1-q4 | serv/w | x | NA | NA | x | NA | NA | NA | NA | [54] |
| WHS | 22,172 | F | V | q1,q3,q5 | serv/d | x | NA | NA | x | NA | NA | x | x | [56] |
| WHS | 39,127 | F | V | q1-q5 | serv/d | x | NA | NA | x | NA | x | NA | NA | [57] |

References

1. Nguyen B, Bauman A, Gale J, Banks E, Kritharides L, Ding D (2016) Fruit and vegetable consumption and all-cause mortality: evidence from a large Australian cohort study. Int J Behav Nutr Phys Act 13:9. doi:10.1186/s12966-016-0334-5

2. Steffen LM, Jacobs DR, Jr., Stevens J, Shahar E, Carithers T, Folsom AR (2003) Associations of whole-grain, refined-grain, and fruit and vegetable consumption with risks of all-cause mortality and incident coronary artery disease and ischemic stroke: the Atherosclerosis Risk in Communities (ARIC) Study. Am J Clin Nutr 78 (3):383-390

3. Michaud DS, Pietinen P, Taylor PR, Virtanen M, Virtamo J, Albanes D (2002) Intakes of fruits and vegetables, carotenoids and vitamins A, E, C in relation to the risk of bladder cancer in the ATBC cohort study. Br J Cancer 87 (9):960-965. doi:10.1038/sj.bjc.6600604

4. Miedema MD, Petrone A, Shikany JM, Greenland P, Lewis CE, Pletcher MJ, Gaziano JM, Djousse L (2015) Association of Fruit and Vegetable Consumption During Early Adulthood With the Prevalence of Coronary Artery Calcium After 20 Years of Follow-Up: The Coronary Artery Risk Development in Young Adults (CARDIA) Study. Circulation 132 (21):1990-1998. doi:10.1161/CIRCULATIONAHA.114.012562

5. Larsson SC, Bergkvist L, Wolk A (2006) Fruit and vegetable consumption and incidence of gastric cancer: a prospective study. Cancer Epidemiol Biomarkers Prev 15 (10):1998-2001. doi:10.1158/1055-9965.EPI-06-0402

6. Johnsen SP, Overvad K, Stripp C, Tjonneland A, Husted SE, Sorensen HT (2003) Intake of fruit and vegetables and the risk of ischemic stroke in a cohort of Danish men and women. Am J Clin Nutr 78 (1):57-64

7. Buchner FL, Bueno-de-Mesquita HB, Linseisen J, Boshuizen HC, Kiemeney LA, Ros MM, Overvad K, Hansen L, Tjonneland A, Raaschou-Nielsen O, Clavel-Chapelon F, Boutron-Ruault MC, Touillaud M, Kaaks R, Rohrmann S, Boeing H, Nothlings U, Trichopoulou A, Zylis D, Dilis V, Palli D, Sieri S, Vineis P, Tumino R, Panico S, Peeters PH, van Gils CH, Lund E, Gram IT, Braaten T, Martinez C, Agudo A, Arriola L, Ardanaz E, Navarro C, Rodriguez L, Manjer J, Wirfalt E, Hallmans G, Rasmuson T, Key TJ, Roddam AW, Bingham S, Khaw KT, Slimani N, Bofetta P, Byrnes G, Norat T, Michaud D, Riboli E (2010) Fruits and vegetables consumption and the risk of histological subtypes of lung cancer in the European Prospective Investigation into Cancer and Nutrition (EPIC). Cancer Causes Control 21 (3):357-371. doi:10.1007/s10552-009-9468-y

8. van Duijnhoven FJ, Bueno-De-Mesquita HB, Ferrari P, Jenab M, Boshuizen HC, Ros MM, Casagrande C, Tjonneland A, Olsen A, Overvad K, Thorlacius-Ussing O, Clavel-Chapelon F, Boutron-Ruault MC, Morois S, Kaaks R, Linseisen J, Boeing H, Nothlings U, Trichopoulou A, Trichopoulos D, Misirli G, Palli D, Sieri S, Panico S, Tumino R, Vineis P, Peeters PH, van Gils CH, Ocke MC, Lund E, Engeset D, Skeie G, Suarez LR, Gonzalez CA, Sanchez MJ, Dorronsoro M, Navarro C, Barricarte A, Berglund G, Manjer J, Hallmans G, Palmqvist R, Bingham SA, Khaw KT, Key TJ, Allen NE, Boffetta P, Slimani N, Rinaldi S, Gallo V, Norat T, Riboli E (2009) Fruit, vegetables, and colorectal cancer risk: the European Prospective Investigation into Cancer and Nutrition. Am J Clin Nutr 89 (5):1441-1452. doi:10.3945/ajcn.2008.27120

9. Leenders M, Boshuizen HC, Ferrari P, Siersema PD, Overvad K, Tjonneland A, Olsen A, Boutron-Ruault MC, Dossus L, Dartois L, Kaaks R, Li K, Boeing H, Bergmann MM, Trichopoulou A, Lagiou P, Trichopoulos D, Palli D, Krogh V, Panico S, Tumino R, Vineis P, Peeters PH, Weiderpass E, Engeset D, Braaten T, Redondo ML, Agudo A, Sanchez MJ, Amiano P, Huerta JM, Ardanaz E, Drake I, Sonestedt E, Johansson I, Winkvist A, Khaw KT, Wareham NJ, Key TJ, Bradbury KE, Johansson M, Licaj I, Gunter MJ, Murphy N, Riboli E, Bueno-de-Mesquita HB (2014) Fruit and vegetable intake and cause-specific mortality in the EPIC study. Eur J Epidemiol 29 (9):639-652. doi:10.1007/s10654-014-9945-9

10. Stefler D, Pikhart H, Kubinova R, Pajak A, Stepaniak U, Malyutina S, Simonova G, Peasey A, Marmot MG, Bobak M (2016) Fruit and vegetable consumption and mortality in Eastern Europe: Longitudinal results from the Health, Alcohol and Psychosocial Factors in Eastern Europe study. Eur J Prev Cardiol 23 (5):493-501. doi:10.1177/2047487315582320

11. Feskanich D, Ziegler RG, Michaud DS, Giovannucci EL, Speizer FE, Willett WC, Colditz GA (2000) Prospective study of fruit and vegetable consumption and risk of lung cancer among men and women. J Natl Cancer Inst 92 (22):1812-1823

12. Bhupathiraju SN, Wedick NM, Pan A, Manson JE, Rexrode KM, Willett WC, Rimm EB, Hu FB (2013) Quantity and variety in fruit and vegetable intake and risk of coronary heart disease. Am J Clin Nutr 98 (6):1514-1523. doi:10.3945/ajcn.113.066381

13. Oyebode O, Gordon-Dseagu V, Walker A, Mindell JS (2014) Fruit and vegetable consumption and all-cause, cancer and CVD mortality: analysis of Health Survey for England data. J Epidemiol Community Health 68 (9):856-862. doi:10.1136/jech-2013-203500

14. Thompson CA, Habermann TM, Wang AH, Vierkant RA, Folsom AR, Ross JA, Cerhan JR (2010) Antioxidant intake from fruits, vegetables and other sources and risk of non-Hodgkin's lymphoma: the Iowa Women's Health Study. Int J Cancer 126 (4):992-1003. doi:10.1002/ijc.24830

15. Kurotani K, Nanri A, Goto A, Mizoue T, Noda M, Kato M, Inoue M, Tsugane S, Japan Public Health Center-based Prospective Study G (2013) Vegetable and fruit intake and risk of type 2 diabetes: Japan Public Health Center-based Prospective Study. Br J Nutr 109 (4):709-717. doi:10.1017/S0007114512001705

16. Takachi R, Inoue M, Ishihara J, Kurahashi N, Iwasaki M, Sasazuki S, Iso H, Tsubono Y, Tsugane S, Group JS (2008) Fruit and vegetable intake and risk of total cancer and cardiovascular disease: Japan Public Health Center-Based Prospective Study. Am J Epidemiol 167 (1):59-70. doi:10.1093/aje/kwm263

17. Rissanen TH, Voutilainen S, Virtanen JK, Venho B, Vanharanta M, Mursu J, Salonen JT (2003) Low intake of fruits, berries and vegetables is associated with excess mortality in men: the Kuopio Ischaemic Heart Disease Risk Factor (KIHD) Study. J Nutr 133 (1):199-204

18. Oude Griep LM, Geleijnse JM, Kromhout D, Ocke MC, Verschuren WM (2010) Raw and processed fruit and vegetable consumption and 10-year coronary heart disease incidence in a population-based cohort study in the Netherlands. PLoS One 5 (10):e13609. doi:10.1371/journal.pone.0013609

19. Bazzano LA, He J, Ogden LG, Loria CM, Vupputuri S, Myers L, Whelton PK (2002) Fruit and vegetable intake and risk of cardiovascular disease in US adults: the first National Health and Nutrition Examination Survey Epidemiologic Follow-up Study. Am J Clin Nutr 76 (1):93-99

20. Wright ME, Park Y, Subar AF, Freedman ND, Albanes D, Hollenbeck A, Leitzmann MF, Schatzkin A (2008) Intakes of fruit, vegetables, and specific botanical groups in relation to lung cancer risk in the NIH-AARP Diet and Health Study. Am J Epidemiol 168 (9):1024-1034. doi:10.1093/aje/kwn212

21. Dubrow R, Darefsky AS, Park Y, Mayne ST, Moore SC, Kilfoy B, Cross AJ, Sinha R, Hollenbeck AR, Schatzkin A, Ward MH (2010) Dietary components related to N-nitroso compound formation: a prospective study of adult glioma. Cancer Epidemiol Biomarkers Prev 19 (7):1709-1722. doi:10.1158/1055-9965.EPI-10-0225

22. Park Y, Subar AF, Kipnis V, Thompson FE, Mouw T, Hollenbeck A, Leitzmann MF, Schatzkin A (2007) Fruit and vegetable intakes and risk of colorectal cancer in the NIH-AARP diet and health study. Am J Epidemiol 166 (2):170-180. doi:10.1093/aje/kwm067

23. Okuda N, Miura K, Okayama A, Okamura T, Abbott RD, Nishi N, Fujiyoshi A, Kita Y, Nakamura Y, Miyagawa N, Hayakawa T, Ohkubo T, Kiyohara Y, Ueshima H, Group NDR (2015) Fruit and vegetable intake and mortality from cardiovascular disease in Japan: a 24-year follow-up of the NIPPON DATA80 Study. Eur J Clin Nutr 69 (4):482-488. doi:10.1038/ejcn.2014.276

24. Genkinger JM, Platz EA, Hoffman SC, Comstock GW, Helzlsouer KJ (2004) Fruit, vegetable, and antioxidant intake and all-cause, cancer, and cardiovascular disease mortality in a community-dwelling population in Washington County, Maryland. Am J Epidemiol 160 (12):1223-1233. doi:10.1093/aje/

25. Shigihara M, Obara T, Nagai M, Sugawara Y, Watanabe T, Kakizaki M, Nishino Y, Kuriyama S, Tsuji I (2014) Consumption of fruits, vegetables, and seaweeds (sea vegetables) and pancreatic cancer risk: the Ohsaki Cohort Study. Cancer Epidemiol 38 (2):129-136. doi:10.1016/j.canep.2014.01.001

26. Kunzmann AT, Coleman HG, Huang WY, Cantwell MM, Kitahara CM, Berndt SI (2016) Fruit and vegetable intakes and risk of colorectal cancer and incident and recurrent adenomas in the PLCO cancer screening trial. Int J Cancer 138 (8):1851-1861. doi:10.1002/ijc.29922

27. Dauchet L, Montaye M, Ruidavets JB, Arveiler D, Kee F, Bingham A, Ferrieres J, Haas B, Evans A, Ducimetiere P, Amouyel P, Dallongeville J (2010) Association between the frequency of fruit and vegetable consumption and cardiovascular disease in male smokers and non-smokers. Eur J Clin Nutr 64 (6):578-586. doi:10.1038/ejcn.2010.46

28. Dauchet L, Ferrieres J, Arveiler D, Yarnell JW, Gey F, Ducimetiere P, Ruidavets JB, Haas B, Evans A, Bingham A, Amouyel P, Dallongeville J (2004) Frequency of fruit and vegetable consumption and coronary heart disease in France and Northern Ireland: the PRIME study. Br J Nutr 92 (6):963-972

29. Rautiainen S, Levitan EB, Mittleman MA, Wolk A (2015) Fruit and vegetable intake and rate of heart failure: a population-based prospective cohort of women. Eur J Heart Fail 17 (1):20-26. doi:10.1002/ejhf.191

30. Larsson SC, Virtamo J, Wolk A (2013) Total and specific fruit and vegetable consumption and risk of stroke: a prospective study. Atherosclerosis 227 (1):147-152. doi:10.1016/j.atherosclerosis.2012.12.022

31. Choi Y, Lee JE, Bae JM, Li ZM, Kim DH, Lee MS, Ahn YO, Shin MH (2015) Vegetable intake, but not fruit intake, is associated with a reduction in the risk of cancer incidence and mortality in middle-aged Korean men. J Nutr 145 (6):1249-1255. doi:10.3945/jn.114.209437

32. Yu D, Zhang X, Gao YT, Li H, Yang G, Huang J, Zheng W, Xiang YB, Shu XO (2014) Fruit and vegetable intake and risk of CHD: results from prospective cohort studies of Chinese adults in Shanghai. Br J Nutr 111 (2):353-362. doi:10.1017/S0007114513002328

33. Zhang X, Shu XO, Xiang YB, Yang G, Li H, Gao J, Cai H, Gao YT, Zheng W (2011) Cruciferous vegetable consumption is associated with a reduced risk of total and cardiovascular disease mortality. Am J Clin Nutr 94 (1):240-246. doi:10.3945/ajcn.110.009340

34. Nunez-Cordoba JM, Alonso A, Beunza JJ, Palma S, Gomez-Gracia E, Martinez-Gonzalez MA (2009) Role of vegetables and fruits in Mediterranean diets to prevent hypertension. Eur J Clin Nutr 63 (5):605-612. doi:10.1038/ejcn.2008.22

35. Diallo A, Deschasaux M, Galan P, Hercberg S, Zelek L, Latino-Martel P, Touvier M (2016) Associations between fruit, vegetable and legume intakes and prostate cancer risk: results from the prospective Supplementation en Vitamines et Mineraux Antioxydants (SU.VI.MAX) cohort. Br J Nutr 115 (9):1579-1585. doi:10.1017/S0007114516000520

36. Lof M, Sandin S, Lagiou P, Trichopoulos D, Adami HO, Weiderpass E (2011) Fruit and vegetable intake and risk of cancer in the Swedish women's lifestyle and health cohort. Cancer Causes Control 22 (2):283-289. doi:10.1007/s10552-010-9696-1

37. Gillman MW, Cupples LA, Gagnon D, Posner BM, Ellison RC, Castelli WP, Wolf PA (1995) Protective effect of fruits and vegetables on development of stroke in men. JAMA 273 (14):1113-1117

38. Flood A, Velie EM, Chaterjee N, Subar AF, Thompson FE, Lacey JV, Jr., Schairer C, Troisi R, Schatzkin A (2002) Fruit and vegetable intakes and the risk of colorectal cancer in the Breast Cancer Detection Demonstration Project follow-up cohort. Am J Clin Nutr 75 (5):936-943

39. Boggs DA, Palmer JR, Wise LA, Spiegelman D, Stampfer MJ, Adams-Campbell LL, Rosenberg L (2010) Fruit and vegetable intake in relation to risk of breast cancer in the Black Women's Health Study. Am J Epidemiol 172 (11):1268-1279. doi:10.1093/aje/kwq293

40. Kobylecki CJ, Afzal S, Davey Smith G, Nordestgaard BG (2015) Genetically high plasma vitamin C, intake of fruit and vegetables, and risk of ischemic heart disease and all-cause mortality: a Mendelian randomization study. Am J Clin Nutr 101 (6):1135-1143. doi:10.3945/ajcn.114.104497

41. McCullough ML, Robertson AS, Chao A, Jacobs EJ, Stampfer MJ, Jacobs DR, Diver WR, Calle EE, Thun MJ (2003) A prospective study of whole grains, fruits, vegetables and colon cancer risk. Cancer Causes Control 14 (10):959-970

42. Bamia C, Lagiou P, Jenab M, Aleksandrova K, Fedirko V, Trichopoulos D, Overvad K, Tjonneland A, Olsen A, Clavel-Chapelon F, Boutron-Ruault MC, Kvaskoff M, Katzke VA, Kuhn T, Boeing H, Nothlings U, Palli D, Sieri S, Panico S, Tumino R, Naccarati A, Bueno-de-Mesquita HB, Peeters PH, Weiderpass E, Skeie G, Quiros JR, Agudo A, Chirlaque MD, Sanchez MJ, Ardanaz E, Dorronsoro M, Ericson U, Nilsson LM, Wennberg M, Khaw KT, Wareham N, Key TJ, Travis RC, Ferrari P, Stepien M, Duarte-Salles T, Norat T, Murphy N, Riboli E, Trichopoulou A (2015) Fruit and vegetable consumption in relation to hepatocellular carcinoma in a multi-centre, European cohort study. Br J Cancer 112 (7):1273-1282. doi:10.1038/bjc.2014.654

43. Michels KB, Edward G, Joshipura KJ, Rosner BA, Stampfer MJ, Fuchs CS, Colditz GA, Speizer FE, Willett WC (2000) Prospective study of fruit and vegetable consumption and incidence of colon and rectal cancers. J Natl Cancer Inst 92 (21):1740-1752

44. Joshipura KJ, Ascherio A, Manson JE, Stampfer MJ, Rimm EB, Speizer FE, Hennekens CH, Spiegelman D, Willett WC (1999) Fruit and vegetable intake in relation to risk of ischemic stroke. JAMA 282 (13):1233-1239

45. Nagura J, Iso H, Watanabe Y, Maruyama K, Date C, Toyoshima H, Yamamoto A, Kikuchi S, Koizumi A, Kondo T, Wada Y, Inaba Y, Tamakoshi A, Group JS (2009) Fruit, vegetable and bean intake and mortality from cardiovascular disease among Japanese men and women: the JACC Study. Br J Nutr 102 (2):285-292. doi:10.1017/S0007114508143586

46. Suzuki R, Iwasaki M, Hara A, Inoue M, Sasazuki S, Sawada N, Yamaji T, Shimazu T, Tsugane S, Japan Public Health Center-based Prospective Study G (2013) Fruit and vegetable intake and breast cancer risk defined by estrogen and progesterone receptor status: the Japan Public Health Center-based Prospective Study. Cancer Causes Control 24 (12):2117-2128. doi:10.1007/s10552-013-0289-7

47. Sauvaget C, Nagano J, Hayashi M, Spencer E, Shimizu Y, Allen N (2003) Vegetables and fruit intake and cancer mortality in the Hiroshima/Nagasaki Life Span Study. Br J Cancer 88 (5):689-694. doi:10.1038/sj.bjc.6600775

48. Breslow RA, Graubard BI, Sinha R, Subar AF (2000) Diet and lung cancer mortality: a 1987 National Health Interview Survey cohort study. Cancer Causes Control 11 (5):419-431

49. Michels KB, Giovannucci E, Chan AT, Singhania R, Fuchs CS, Willett WC (2006) Fruit and vegetable consumption and colorectal adenomas in the Nurses' Health Study. Cancer Res 66 (7):3942-3953. doi:10.1158/0008-5472.CAN-05-3637

50. Farvid MS, Chen WY, Michels KB, Cho E, Willett WC, Eliassen AH (2016) Fruit and vegetable consumption in adolescence and early adulthood and risk of breast cancer: population based cohort study. BMJ 353:i2343. doi:10.1136/bmj.i2343

51. Kirsh VA, Peters U, Mayne ST, Subar AF, Chatterjee N, Johnson CC, Hayes RB, Prostate LC, Ovarian Cancer Screening T (2007) Prospective study of fruit and vegetable intake and risk of prostate cancer. J Natl Cancer Inst 99 (15):1200-1209. doi:10.1093/jnci/djm065

52. Villegas R, Shu XO, Gao YT, Yang G, Elasy T, Li H, Zheng W (2008) Vegetable but not fruit consumption reduces the risk of type 2 diabetes in Chinese women. J Nutr 138 (3):574-580

53. Nakamura K, Nagata C, Oba S, Takatsuka N, Shimizu H (2008) Fruit and vegetable intake and mortality from cardiovascular disease are inversely associated in Japanese women but not in men. J Nutr 138 (6):1129-1134

54. Strandhagen E, Hansson PO, Bosaeus I, Isaksson B, Eriksson H (2000) High fruit intake may reduce mortality among middle-aged and elderly men. The Study of Men Born in 1913. Eur J Clin Nutr 54 (4):337-341

55. Lai HT, Threapleton DE, Day AJ, Williamson G, Cade JE, Burley VJ (2015) Fruit intake and cardiovascular disease mortality in the UK Women's Cohort Study. Eur J Epidemiol 30 (9):1035-1048. doi:10.1007/s10654-015-0050-5

56. Lin J, Zhang SM, Cook NR, Rexrode KM, Liu S, Manson JE, Lee IM, Buring JE (2005) Dietary intakes of fruit, vegetables, and fiber, and risk of colorectal cancer in a prospective cohort of women (United States). Cancer Causes Control 16 (3):225-233. doi:10.1007/s10552-004-4025-1

57. Liu S, Manson JE, Lee IM, Cole SR, Hennekens CH, Willett WC, Buring JE (2000) Fruit and vegetable intake and risk of cardiovascular disease: the Women's Health Study. Am J Clin Nutr 72 (4):922-928

58. Bendinelli B, Masala G, Saieva C, Salvini S, Calonico C, Sacerdote C, Agnoli C, Grioni S, Frasca G, Mattiello A, Chiodini P, Tumino R, Vineis P, Palli D, Panico S (2011) Fruit, vegetables, and olive oil and risk of coronary heart disease in Italian women: the EPICOR Study. Am J Clin Nutr 93 (2):275-283. doi:10.3945/ajcn.110.000521

59. Liu Y, Sobue T, Otani T, Tsugane S (2004) Vegetables, fruit consumption and risk of lung cancer among middle-aged Japanese men and women: JPHC study. Cancer Causes Control 15 (4):349-357. doi:10.1023/B:CACO.0000027507.22124.20

60. Takachi R, Inoue M, Sawada N, Iwasaki M, Sasazuki S, Ishihara J, Tsubono Y, Tsugane S, Japan Public Health Center-Based Prospective Study G (2010) Fruits and vegetables in relation to prostate cancer in Japanese men: the Japan Public Health Center-Based Prospective Study. Nutr Cancer 62 (1):30-39. doi:10.1080/01635580903191502

61. Nothlings U, Wilkens LR, Murphy SP, Hankin JH, Henderson BE, Kolonel LN (2007) Vegetable intake and pancreatic cancer risk: the multiethnic cohort study. Am J Epidemiol 165 (2):138-147. doi:10.1093/aje/kwj366
