# Supplementary material for: Health risk factors associated with meat, fruit and vegetable consumption in cohort studies: A comprehensive meta-analysis
Source: PLoS One. 2017 Aug 29;12(8):e0183787. doi: 10.1371/journal.pone.0183787 (PMC5574618; doi:10.1371/journal.pone.0183787)
Supplement: S4 Table — NA, not applicable. (DOCX) [file pone.0183787.s004.docx]

**Supplementary Table 4.** Summary associations between selected variables and red meat consumption, by sexes. NA, not applicable.

|  | Men |  |  | Women |  |  |
| --- | --- | --- | --- | --- | --- | --- |
| Variables | No. of cohorts | No. of individuals | Slope per 100 g/d (95% CI) | No. of cohorts | No. of individuals | Slope per 100 g/d (95% CI) |
| BMI (mean/median) | 8 | 623,708 | 0.93 (0.31, 1.54) | 9 | 884,404 | 1.6 (0.71, 2.5) |
| BMI >30 (%) | 2 | 52,441 | 8.32 (7.18, 9.45) | 0 | 0 | NA |
| BMI >25 (%) | 3 | 90,903 | 7.91 (7, 8.82) | 1 | 42,196 | 5.92 (1.57, 10.26) |
| Current smokers (%) | 10 | 707,565 | 6.14 (3.15, 9.13) | 10 | 954,122 | 5.77 (3.44, 8.1) |
| Former smokers (%) | 7 | 563,168 | -3.03 (-5.86, -0.19) | 6 | 666,211 | -4.35 (-7.51, -1.19) |
| Ever smokers (%) | 7 | 563,168 | 3.65 (-0.25, 7.54) | 6 | 666,211 | 2.86 (0.52, 5.2) |
| Never smokers (%) | 6 | 524,706 | -5.1 (-8.7, -1.51) | 5 | 624,015 | -5.54 (-7.59, -3.49) |
| High physical activity (%) | 6 | 546,141 | -3.09 (-7.97, 1.79) | 5 | 651,611 | -7.31 (-11.72, -2.91) |
| Vocational/High school (%) | 4 | 236,743 | -5.78 (-12.76, 1.19) | 3 | 415,331 | -7.32 (-18.4, 3.76) |
| College/University (%) | 4 | 529,025 | -9.13 (-15.46, -2.81) | 4 | 648,753 | -11.39 (-16.65, -6.12) |
| Alcohol (g/d, mean/median) | 5 | 519,891 | 2.27 (-3.18, 7.72) | 6 | 772,258 | 0.88 (-0.22, 1.98) |
| Fruit (g/d, mean/median) | 6 | 553,527 | -65.39 (-124.77, -6.01) | 6 | 714,173 | -81.05 (-167.43, 5.33) |
| Vegetable (g/d, mean/median) | 6 | 553,527 | -7.36 (-35.46, 20.74) | 6 | 714,173 | -3.38 (-54, 47.23) |
| Fruit+vegetables (g/d, mean/median) | 1 | 21,461 | -55.95 (-137.33, 25.42) | 2 | 99,879 | 26.63 (-22.96, 76.23) |
