# Supplementary material for: Health risk factors associated with meat, fruit and vegetable consumption in cohort studies: A comprehensive meta-analysis
Source: PLoS One. 2017 Aug 29;12(8):e0183787. doi: 10.1371/journal.pone.0183787 (PMC5574618; doi:10.1371/journal.pone.0183787)
Supplement: S5 Table — NA, not applicable. (DOCX) [file pone.0183787.s005.docx]

**Supplementary Table 5.** Summary associations between selected variables and red meat consumption, by geographical region. NA, not applicable.

|  | Europe |  |  | US |  |  | Asia |  |  |
| --- | --- | --- | --- | --- | --- | --- | --- | --- | --- |
| Variables | No. of cohorts | No. of individuals | Slope per 100 g/d (95% CI) | No. of cohorts | No. of individuals | Slope per 100 g/d (95% CI) | No. of cohorts | No. of individuals | Slope per 100 g/d (95% CI) |
| BMI (mean/median) | 5 | 552,000 | 0.55 (0.27, 0.82) | 8 | 1,056,260 | 1.8 (1.03, 2.58) | 1 | 42,403 | 0.19 (-0.01, 0.39) |
| BMI >30 (%) | 0 | 0 | NA | 2 | 52,441 | 8.32 (7.18, 9.45) | 0 | 0 | NA |
| BMI >25 (%) | 0 | 0 | NA | 2 | 52,441 | 7.63 (6.38, 8.88) | 1 | 80,658 | 7.29 (4.61, 9.97) |
| Current smokers (%) | 5 | 601,837 | 6.2 (3.12, 9.28) | 8 | 979,909 | 7.39 (5.46, 9.32) | 2 | 123,061 | -0.28 (-2.92, 2.37) |
| Former smokers (%) | 3 | 530,132 | -1.45 (-6.31, 3.41) | 4 | 619,306 | -4.48 (-7.94, -1.02) | 1 | 80,658 | -0.81 (-3.2, 1.58) |
| Ever smokers (%) | 3 | 530,132 | 7.29 (0.66, 13.93) | 4 | 619,306 | 4.22 (1.18, 7.26) | 1 | 80,658 | -1.85 (-8.03, 4.33) |
| Never smokers (%) | 3 | 530,132 | -8.29 (-14.85, -1.73) | 4 | 619,306 | -5.27 (-8.03, -2.51) | 0 | 0 | NA |
| High physical activity (%) | 2 | 529,415 | -0.12 (-4.34, 4.09) | 5 | 685,409 | -8.4 (-11.59, -5.21) | 1 | 42,403 | -0.36 (-2.24, 1.52) |
| Vocational/high school (%) | 2 | 529,415 | -1.95 (-5.9, 2) | 2 | 122,659 | -12.43 (-20.86, -4) | 0 | 0 | NA |
| College/university (%) | 3 | 550,132 | -5.47 (-8.06, -2.88) | 2 | 627,646 | -15.05 (-17.38, -12.72) | 0 | 0 | NA |
| Alcohol (g/d, mean/median) | 5 | 601,837 | 4.13 (1.93, 6.32) | 6 | 807,532 | -0.57 (-3.18, 2.04) | 0 | 0 | NA |
| Fruit (g/d, mean/median) | 4 | 550,849 | -5.33 (-34.11, 23.45) | 5 | 717,568 | -105 (-168.38, -41.61) | 1 | 42,403 | 28.48 (17.69, 39.26) |
| Vegetable (g/d, mean/median) | 4 | 550,849 | 17.12 (-20, 54.25) | 5 | 717,568 | -15.17 (-49.22, 18.89) | 1 | 42,403 | 12.93 (7.87, 17.98) |
| Fruit+vegetable (g/d, mean/median) | 1 | 74,645 | 43.57 (-96.5, 183.64) | 3 | 121,340 | -0.89 (-62.01, 60.23) | 0 | 0 | NA |
