# Supplementary material for: Health risk factors associated with meat, fruit and vegetable consumption in cohort studies: A comprehensive meta-analysis
Source: PLoS One. 2017 Aug 29;12(8):e0183787. doi: 10.1371/journal.pone.0183787 (PMC5574618; doi:10.1371/journal.pone.0183787)
Supplement: S6 Table — (DOCX) [file pone.0183787.s006.docx]

**Supplementary Table 6.** Summary associations between selected variables and processed meat consumption.

| Variables | No. of studies | No. of datasets | No. of cohorts | No. of individuals | Intercept (95% CI) | Slope per 100 g/d (95% CI) |
| --- | --- | --- | --- | --- | --- | --- |
| BMI (mean/median) | 5 | 7 | 7 | 283,645 | 25.11 (23.19, 27.03) | 4.55 (1.6, 7.5) |
| Current smokers (%) | 4 | 6 | 6 | 222,212 | 17.87 (9.87, 25.88) | 25.45 (12.7, 38.2) |
| Former smokers (%) | 2 | 4 | 4 | 140,561 | 29.99 (20.55, 39.44) | -14.46 (-27.42, -1.5) |
| Ever smokers (%) | 3 | 5 | 5 | 201,994 | 47.93 (40.3, 55.56) | 13.72 (-4.58, 32.02) |
| College/University (%) | 4 | 5 | 4 | 688,854 | 32.35 (19.64, 45.05) | -17.12 (-26.79, -7.46) |
| Alcohol (g/d, mean/median) | 4 | 6 | 6 | 281,644 | 6.44 (4.06, 8.82) | 6.98 (-0.94, 14.9) |
| Fruit (g/d, mean/median) | 3 | 3 | 3 | 100,469 | 138.88 (95.95, 181.82) | -30.64 (-90.53, 29.24) |
| Fruit+vegetable (g/d, mean/median) | 3 | 6 | 5 | 728,946 | 621.42 (544.5, 698.33) | -283.02 (-385.38, -180.66) |
