# Supplementary material for: Health risk factors associated with meat, fruit and vegetable consumption in cohort studies: A comprehensive meta-analysis
Source: PLoS One. 2017 Aug 29;12(8):e0183787. doi: 10.1371/journal.pone.0183787 (PMC5574618; doi:10.1371/journal.pone.0183787)
Supplement: S7 Table — (DOCX) [file pone.0183787.s007.docx]

**Supplementary Table 7.** Summary associations between selected variables and processed meat consumption, by sexes.

|  | Men |  |  | Women |  |  |
| --- | --- | --- | --- | --- | --- | --- |
| Variables | No. of cohorts | No. of individuals | Slope per 100 g/d (95% CI) | No. of cohorts | No. of individuals | Slope per 100 g/d (95% CI) |
| BMI (mean/median) | 2 | 65,445 | 1.49 (-0.58, 3.56) | 4 | 216,199 | 4.8 (0.98, 8.62) |
| Current smokers (%) | 2 | 65,445 | 17.44 (-15.95, 50.83) | 3 | 154,766 | 24.56 (13.29, 35.83) |
| Former smokers (%) | 1 | 28,410 | 3.65 (-16.88, 24.18) | 2 | 110,150 | -24.25 (-27.04, -21.45) |
| Ever smokers (%) | 1 | 28,410 | 38.14 (2.8, 73.49) | 3 | 171,583 | -0.27 (-12.81, 12.27) |
| College/university (%) | 2 | 359,382 | -20.65 (-34.73, -6.57) | 3 | 329,472 | -14.77 (-29.56, 0.03) |
| Alcohol (g/d, mean/median) | 2 | 65,445 | 9.22 (-6.12, 24.56) | 4 | 216,199 | 5.86 (-4.76, 16.48) |
| Fruit (g/d, mean/median) | 1 | 37,035 | 23.03 (-1.22, 47.29) | 1 | 61,433 | -37.42 (-95.15, 20.32) |
| Fruit+vegetables (g/d, mean/median) | 2 | 350,757 | -359.05 (-377.96, -340.14) | 4 | 378,189 | -244.16 (-385.95, -102.38) |
