# Supplementary material for: Health risk factors associated with meat, fruit and vegetable consumption in cohort studies: A comprehensive meta-analysis
Source: PLoS One. 2017 Aug 29;12(8):e0183787. doi: 10.1371/journal.pone.0183787 (PMC5574618; doi:10.1371/journal.pone.0183787)
Supplement: S8 Table — (DOCX) [file pone.0183787.s008.docx]

**Supplementary Table 8.** Summary associations between selected variables and processed meat consumption, by geographical region. No data from Asian cohorts were available. NA, not applicable.

|  | Europe |  |  | US |  |  |
| --- | --- | --- | --- | --- | --- | --- |
| Variables | No. of cohorts | No. of individuals | Slope per 100 g/d (95% CI) | No. of cohorts | No. of individuals | Slope per 100 g/d (95% CI) |
| BMI (mean/median) | 3 | 143,084 | 1.47 (0.47, 2.47) | 4 | 140,561 | 6.87 (3.14, 10.61) |
| Current smokers (%) | 2 | 81,651 | 6.75 (-5.69, 19.19) | 4 | 140,561 | 34.67 (28.38, 40.95) |
| Former smokers (%) | 0 | 0 | NA | 4 | 140,561 | -14.46 (-27.42, -1.5) |
| Ever smokers (%) | 1 | 61,433 | -12.89 (-13.24, -12.54) | 4 | 140,561 | 20.37 (3.8, 36.94) |
| College/university (%) | 3 | 143,084 | -9.36 (-13.89, -4.83) | 1 | 545,770 | -28.77 (-30.64, -26.91) |
| Alcohol (g/d, mean/median) | 3 | 143,084 | 7.86 (-6.13, 21.85) | 3 | 138,560 | 6.1 (-4.62, 16.82) |
| Fruit (g/d, mean/median) | 2 | 98,468 | -7.17 (-66.46, 52.12) | 1 | 2,001 | -82.23 (-198.35, 33.89) |
| Fruit+vegetable (g/d, mean/median) | 1 | 44,616 | -244.94 (-421.46, -68.43) | 4 | 684,330 | -290.44 (-414.68, -166.2) |
