# Supplementary material for: Health risk factors associated with meat, fruit and vegetable consumption in cohort studies: A comprehensive meta-analysis
Source: PLoS One. 2017 Aug 29;12(8):e0183787. doi: 10.1371/journal.pone.0183787 (PMC5574618; doi:10.1371/journal.pone.0183787)
Supplement: S9 Table — (DOCX) [file pone.0183787.s009.docx]

**Supplementary Table 9.** Summary associations between selected variables and total meat consumption.

| Variables | No. of studies | No. of datasets | No. of cohorts | No. of individuals | Intercept (95% CI) | Slope per 100 g/d (95% CI) |
| --- | --- | --- | --- | --- | --- | --- |
| BMI (mean/median) | 10 | 13 | 10 | 424,638 | 23.78 (23.32, 24.25) | 0.58 (0.22, 0.94) |
| BMI >30 (%) | 1 | 2 | 1 | 322,846 | 10.59 (6.75, 14.43) | 7.6 (6.56, 8.65) |
| BMI >25 (%) | 2 | 3 | 2 | 364,681 | 43.96 (34.14, 53.78) | 1.53 (-15.23, 18.29) |
| Former smokers (%) | 5 | 6 | 5 | 469,102 | 36.5 (25.12, 47.88) | -0.89 (-3.45, 1.68) |
| Ever smokers (%) | 5 | 6 | 5 | 469,102 | 56.6 (51.51, 61.68) | -2.34 (-9.96, 5.28) |
| Never smokers (%) | 5 | 6 | 5 | 469,102 | 42.26 (36.77, 47.75) | 2.37 (-5.24, 9.97) |
| High physical activity (%) | 5 | 8 | 5 | 453,020 | 41.1 (28.66, 53.53) | -1.92 (-8.09, 4.26) |
| Vocational/high school (%) | 2 | 3 | 2 | 52,547 | 37.46 (30.81, 44.11) | 3.89 (-14.92, 22.7) |
| College/university (%) | 6 | 9 | 6 | 554,575 | 25.87 (15.47, 36.26) | -0.68 (-7.03, 5.67) |
| Alcohol (g/d, mean/median) | 8 | 10 | 8 | 327,064 | 8.72 (2.16, 15.28) | -0.05 (-3.41, 3.31) |
| Fruit (g/d, mean/median) | 5 | 7 | 5 | 152,362 | 218.12 (136.12, 300.13) | -1.33 (-26.97, 24.3) |
