# Supplementary material for: Health risk factors associated with meat, fruit and vegetable consumption in cohort studies: A comprehensive meta-analysis
Source: PLoS One. 2017 Aug 29;12(8):e0183787. doi: 10.1371/journal.pone.0183787 (PMC5574618; doi:10.1371/journal.pone.0183787)
Supplement: S10 Table — (DOCX) [file pone.0183787.s010.docx]

**Supplementary Table 10.** Summary associations between selected variables and total meat consumption, by sexes.

|  | Men |  |  | Women |  |  |
| --- | --- | --- | --- | --- | --- | --- |
| Variables | No. of cohorts | No. of individuals | Slope per 100 g/d (95% CI) | No. of cohorts | No. of individuals | Slope per 100 g/d (95% CI) |
| BMI (mean/median) | 5 | 82,724 | 0.49 (-0.08, 1.06) | 6 | 241,385 | 0.77 (0.16, 1.39) |
| BMI >30 (%) | 1 | 187,265 | 8.14 (7.7, 8.57) | 1 | 135,581 | 7.07 (5.62, 8.52) |
| BMI >25 (%) | 1 | 187,265 | 10.22 (8.02, 12.42) | 1 | 135,581 | 9.95 (6.59, 13.31) |
| Former smokers (%) | 2 | 191,157 | -0.23 (-0.87, 0.4) | 1 | 135,581 | 2 (1.31, 2.68) |
| Ever smokers (%) | 2 | 191,157 | 1.18 (0.41, 1.95) | 1 | 135,581 | 3.03 (2.73, 3.33) |
| Never smokers (%) | 2 | 191,157 | -1.13 (-1.98, -0.27) | 1 | 135,581 | -2.99 (-3.39, -2.58) |
| High physical activity (%) | 4 | 238,672 | -0.21 (-4.36, 3.94) | 3 | 172,513 | 2.1 (-5.13, 9.32) |
| Vocational/high school (%) | 1 | 4,998 | 1.42 (-0.06, 2.89) | 1 | 5,714 | -11.35 (-18.52, -4.19) |
| College/university (%) | 3 | 212,729 | -4.52 (-15.43, 6.39) | 4 | 218,009 | -2.16 (-9.2, 4.88) |
| Alcohol (g/d, mean/median) | 4 | 55,299 | 0.74 (-6.41, 7.89) | 4 | 171,236 | -1.62 (-6.87, 3.63) |
| Fruit (g/d, mean/median) | 3 | 51,407 | 11.64 (-39.19, 62.47) | 3 | 82,428 | -13.65 (-51.16, 23.85) |
| Vegetable (g/d, mean/median) | 4 | 78,832 | 21.64 (-20.4, 63.69) | 4 | 118,852 | -5.51 (-47.68, 36.66) |
