# Supplementary material for: Health risk factors associated with meat, fruit and vegetable consumption in cohort studies: A comprehensive meta-analysis
Source: PLoS One. 2017 Aug 29;12(8):e0183787. doi: 10.1371/journal.pone.0183787 (PMC5574618; doi:10.1371/journal.pone.0183787)
Supplement: S11 Table — NA, not applicable. (DOCX) [file pone.0183787.s011.docx]

**Supplementary Table 11.** Summary associations between selected variables and total meat consumption, by geographical region. NA, not applicable.

|  | Europe |  |  | US |  |  | Asia |  |  |
| --- | --- | --- | --- | --- | --- | --- | --- | --- | --- |
| Variables | No. of cohorts | No. of individuals | Slope per 100 g/d (95% CI) | No. of cohorts | No. of individuals | Slope per 100 g/d (95% CI) | No. of cohorts | No. of individuals | Slope per 100 g/d (95% CI) |
| BMI (mean/median) | 5 | 170,909 | 0.84 (0.28, 1.39) | 3 | 138,196 | 0.82 (0.49, 1.15) | 2 | 115,533 | 0 (-0.49, 0.5) |
| BMI >30 (%) | 0 | 0 | NA | 1 | 322,846 | 7.6 (6.56, 8.65) | 0 | 0 | NA |
| BMI >25 (%) | 0 | 0 | NA | 1 | 322,846 | 10.08 (9.81, 10.36) | 1 | 41,835 | -15.57 (-20.19, -10.95) |
| Former smokers (%) | 2 | 100,529 | -0.28 (-5.27, 4.71) | 2 | 326,738 | 0.51 (-0.99, 2.01) | 1 | 41,835 | -6.38 (-20.2, 7.44) |
| Ever smokers (%) | 2 | 100,529 | 0.99 (-1.61, 3.59) | 2 | 326,738 | 1.81 (0.49, 3.13) | 1 | 41,835 | -22.73 (-94.08, 48.61) |
| Never smokers (%) | 2 | 100,529 | -0.99 (-3.59, 1.61) | 2 | 326,738 | -1.76 (-3.07, -0.45) | 1 | 41,835 | 22.73 (-48.61, 94.08) |
| High physical activity (%) | 2 | 36,655 | -0.49 (-1.64, 0.66) | 1 | 322,846 | -3.58 (-4.8, -2.37) | 2 | 93,519 | -1.99 (-20.63, 16.65) |
| Vocational/high school (%) | 1 | 10,712 | -4.99 (-17.7, 7.72) | 0 | 0 | NA | 1 | 41,835 | 21.61 (16.78, 26.45) |
| College/university (%) | 2 | 92,714 | -9.89 (-17.76, -2.02) | 2 | 368,342 | -0.57 (-2.06, 0.91) | 2 | 93,519 | 8.41 (-1.56, 18.37) |
| Alcohol (g/d, mean/median) | 4 | 137,184 | 3.6 (1.09, 6.12) | 3 | 138,196 | -0.12 (-0.79, 0.56) | 1 | 51,684 | -9.1 (-9.47, -8.72) |
| Fruit (g/d, mean/median) | 3 | 55,182 | -11.36 (-46.03, 23.31) | 1 | 45,496 | -28.09 (-48.83, -7.35) | 1 | 51,684 | 31.92 (16.67, 47.17) |
| Vegetable (g/d, mean/median) | 3 | 55,182 | 13.81 (-29.6, 57.23) | 1 | 45,496 | 26.78 (22.77, 30.78) | 2 | 115,533 | 2.05 (-43.03, 47.13) |
