# Supplementary material for: Health risk factors associated with meat, fruit and vegetable consumption in cohort studies: A comprehensive meta-analysis
Source: PLoS One. 2017 Aug 29;12(8):e0183787. doi: 10.1371/journal.pone.0183787 (PMC5574618; doi:10.1371/journal.pone.0183787)
Supplement: S12 Table — (DOCX) [file pone.0183787.s012.docx]

**Supplementary Table 12.** Summary associations between selected variables and fruit+vegetable consumption, by sexes.

|  | Men |  |  | Women |  |  |
| --- | --- | --- | --- | --- | --- | --- |
| Variables | No. of cohorts | No. of individuals | Slope per 100 g/d (95% CI) | No. of cohorts | No. of individuals | Slope per 100 g/d (95% CI) |
| BMI (mean/median) | 12 | 602,247 | 0.02 (-0.03, 0.07) | 8 | 760,424 | -0.01 (-0.05, 0.04) |
| BMI >30 (%) | 1 | 2,641 | -0.5 (-1.5, 0.5) | 1 | 44,838 | -0.18 (-0.95, 0.6) |
| BMI >25 (%) | 3 | 55,287 | -0.53 (-1.26, 0.21) | 3 | 103,614 | 0.13 (-0.8, 1.07) |
| Current smokers (%) | 10 | 598,866 | -4.05 (-5.62, -2.48) | 9 | 797,233 | -2.69 (-3.54, -1.83) |
| Former smokers (%) | 6 | 488,516 | 2.1 (0.66, 3.53) | 6 | 685,638 | 0.68 (-0.01, 1.38) |
| Ever smokers (%) | 7 | 524,425 | -2.33 (-3.34, -1.31) | 7 | 727,620 | -2.15 (-2.99, -1.32) |
| Never smokers (%) | 7 | 524,425 | 2.55 (1.69, 3.4) | 7 | 727,620 | 2.11 (1.29, 2.93) |
| High physical activity (%) | 7 | 356,290 | 2.38 (0.18, 4.59) | 5 | 484,471 | 1.02 (0.44, 1.6) |
| Low physical activity (%) | 4 | 186,971 | -1.23 (-2.15, -0.3) | 2 | 119,738 | -1.53 (-2.52, -0.53) |
| Vocational/high school (%) | 6 | 116,947 | -0.01 (-1.98, 1.96) | 4 | 136,563 | 0.15 (-2.51, 2.8) |
| College/university (%) | 9 | 781,039 | 2.34 (1.39, 3.29) | 7 | 622,602 | 2.7 (1.72, 3.69) |
| Alcohol (g/d, mean/median) | 7 | 527,462 | -0.42 (-1.15, 0.3) | 4 | 625,511 | 0.03 (-0.18, 0.24) |
| Red meat (g/d, mean/median) | 4 | 436,021 | -5.7 (-17.47, 6.08) | 5 | 413,728 | -2.5 (-10.65, 5.64) |
| Processed meat (g/d, mean/median) | 2 | 367,685 | -2.32 (-8.53, 3.88) | 2 | 260,087 | -1.07 (-5.69, 3.54) |
