# Supplementary material for: Health risk factors associated with meat, fruit and vegetable consumption in cohort studies: A comprehensive meta-analysis
Source: PLoS One. 2017 Aug 29;12(8):e0183787. doi: 10.1371/journal.pone.0183787 (PMC5574618; doi:10.1371/journal.pone.0183787)
Supplement: S13 Table — NA, not applicable. (DOCX) [file pone.0183787.s013.docx]

**Supplementary Table 13.** Summary associations between selected variables and fruit+vegetable consumption, by geographical region. NA, not applicable.

|  | Europe |  |  | US |  |  | Asia |  |  |
| --- | --- | --- | --- | --- | --- | --- | --- | --- | --- |
| Variables | No. of cohorts | No. of individuals | Slope per 100 g/d (95% CI) | No. of cohorts | No. of individuals | Slope per 100 g/d (95% CI) | No. of cohorts | No. of individuals | Slope per 100 g/d (95% CI) |
| BMI (mean/median) | 11 | 696,408 | -0.01 (-0.06, 0.04) | 9 | 713,961 | -0.07 (-0.14, 0.01) | 5 | 208,084 | 0.07 (0.03, 0.11) |
| BMI >30 (%) | 4 | 132,038 | 0.03 (-0.57, 0.63) | 1 | 6,151 | 0.06 (-0.21, 0.32) | 0 | 0 | NA |
| BMI >25 (%) | 3 | 113,377 | 0.03 (-1.26, 1.32) | 1 | 6,151 | 0.11 (-1.27, 1.49) | 2 | 110,750 | -0.27 (-0.55, 0) |
| Current smokers (%) | 12 | 761,365 | -3.77 (-5.03, -2.51) | 9 | 719,280 | -2.73 (-3.35, -2.1) | 4 | 177,387 | -3.22 (-5.75, -0.69) |
| Former smokers (%) | 7 | 637,319 | 0.99 (-0.32, 2.29) | 6 | 696,226 | 0.38 (-0.75, 1.51) | 2 | 42,591 | 1.69 (-1.23, 4.61) |
| Ever smokers (%) | 7 | 637,319 | -2.69 (-3.52, -1.86) | 6 | 696,226 | -2.06 (-3.37, -0.76) | 3 | 120,482 | -2.45 (-3.45, -1.45) |
| Never smokers (%) | 7 | 637,319 | 2.68 (1.85, 3.52) | 6 | 696,226 | 2.33 (0.96, 3.71) | 3 | 120,482 | 2.46 (1.45, 3.47) |
| High physical activity (%) | 4 | 529,382 | 2.48 (-0.69, 5.65) | 4 | 365,023 | 1.74 (0.66, 2.81) | 2 | 81,296 | 1.66 (0.79, 2.54) |
| Low physical activity (%) | 5 | 170,930 | -1.57 (-2.78, -0.36) | 4 | 361,691 | -2.3 (-3.81, -0.78) | 0 | 0 | NA |
| Vocational/high school (%) | 5 | 561,894 | 0.55 (-1.48, 2.59) | 3 | 72,220 | -0.96 (-3.78, 1.87) | 3 | 167,655 | 1.36 (0.25, 2.47) |
| College/university (%) | 10 | 782,960 | 2.48 (1.47, 3.5) | 4 | 1,113,290 | 2.54 (1.73, 3.36) | 4 | 182,774 | 1.12 (0.21, 2.03) |
| Alcohol (g/d, mean/median) | 6 | 560,045 | -0.46 (-1.14, 0.22) | 5 | 666,856 | -0.62 (-1.34, 0.09) | 1 | 15,119 | 0.8 (0.45, 1.14) |
| Red meat (g/d, mean/median) | 2 | 526,122 | 0.93 (0.5, 1.36) | 5 | 751,979 | -7.93 (-16.61, 0.75) | 3 | 155,544 | 1.39 (-0.56, 3.34) |
| Processed meat (g/d, mean/median) | 3 | 533,153 | 0.29 (-1.8, 2.38) | 2 | 603,544 | -4.18 (-6.11, -2.24) | 0 | 0 | NA |
