# Supplementary material for: Health risk factors associated with meat, fruit and vegetable consumption in cohort studies: A comprehensive meta-analysis
Source: PLoS One. 2017 Aug 29;12(8):e0183787. doi: 10.1371/journal.pone.0183787 (PMC5574618; doi:10.1371/journal.pone.0183787)
Supplement: S14 Table — (DOCX) [file pone.0183787.s014.docx]

**Supplementary Table 14.** Summary associations between selected variables and fruit consumption.

| Variables | No. of studies | No. of datasets | No. of cohorts | No. of individuals | Intercept (95% CI) | Slope per 100 g/d (95% CI) |
| --- | --- | --- | --- | --- | --- | --- |
| BMI (mean/median) | 16 | 18 | 16 | 718,985 | 24.49 (23.39, 25.6) | 0.08 (-0.28, 0.45) |
| BMI >30 (%) | 3 | 3 | 3 | 701,959 | 17.81 (10.66, 24.96) | 0.41 (-1.11, 1.93) |
| BMI >25 (%) | 3 | 3 | 3 | 701,959 | 42.87 (21.41, 64.32) | 0.97 (-0.95, 2.89) |
| Current smokers (%) | 13 | 16 | 14 | 1,153,960 | 26.02 (17.59, 34.45) | -3.88 (-7.24, -0.53) |
| Former smokers (%) | 5 | 7 | 5 | 673,438 | 31.54 (18.55, 44.53) | -0.96 (-3.37, 1.44) |
| Ever smokers (%) | 10 | 11 | 10 | 888,396 | 56.87 (45.8, 67.93) | -4.17 (-7.75, -0.58) |
| Never smokers (%) | 10 | 11 | 10 | 888,396 | 37.65 (24.87, 50.44) | 8.38 (0.94, 15.82) |
| High physical activity (%) | 8 | 8 | 8 | 948,842 | 30.16 (18.53, 41.8) | 4.08 (1.54, 6.62) |
| Low physical activity (%) | 3 | 3 | 3 | 676,895 | 32.4 (13.55, 51.24) | -2.67 (-6.56, 1.22) |
| Vocational/high school (%) | 4 | 4 | 4 | 619,988 | 26.61 (14.04, 39.18) | 8.68 (-6.38, 23.75) |
| College/university (%) | 9 | 11 | 9 | 1,002,204 | 22.06 (13.64, 30.47) | 6.12 (0.32, 11.92) |
| Alcohol (g/d, mean/median) | 8 | 9 | 8 | 318,564 | 15.8 (4.65, 26.94) | -3.28 (-6.1, -0.46) |
| Red meat (g/d, mean/median) | 6 | 7 | 6 | 249,731 | 64.9 (39.39, 90.4) | -3.1 (-4.77, -1.43) |
