# Supplementary material for: Health risk factors associated with meat, fruit and vegetable consumption in cohort studies: A comprehensive meta-analysis
Source: PLoS One. 2017 Aug 29;12(8):e0183787. doi: 10.1371/journal.pone.0183787 (PMC5574618; doi:10.1371/journal.pone.0183787)
Supplement: S15 Table — NA, not applicable. (DOCX) [file pone.0183787.s015.docx]

**Supplementary Table 15.** Summary associations between selected variables and fruit consumption, by sexes. NA, not applicable.

|  | Men |  |  | Women |  |  |
| --- | --- | --- | --- | --- | --- | --- |
| Variables | No. of cohorts | No. of individuals | Slope per 100 g/d (95% CI) | No. of cohorts | No. of individuals | Slope per 100 g/d (95% CI) |
| BMI (mean/median) | 4 | 98,973 | -0.01 (-0.11, 0.1) | 10 | 421,110 | -0.16 (-0.48, 0.15) |
| BMI >30 (%) | 0 | 0 | NA | 1 | 64,191 | 0.31 (-0.55, 1.17) |
| BMI >25 (%) | 0 | 0 | NA | 1 | 64,191 | 1.4 (-0.02, 2.81) |
| Current smokers (%) | 4 | 74,856 | -8.32 (-17.62, 0.98) | 8 | 343,311 | -3.17 (-4.59, -1.75) |
| Former smokers (%) | 2 | 50,918 | 0.94 (-2.19, 4.06) | 3 | 97,181 | -0.23 (-0.45, -0.02) |
| Ever smokers (%) | 1 | 37,563 | -1.73 (-3.3, -0.16) | 5 | 204,613 | -6.18 (-12.94, 0.58) |
| Never smokers (%) | 1 | 37,563 | 1.73 (0.16, 3.3) | 5 | 204,613 | 6.18 (-0.58, 12.94) |
| High physical activity (%) | 0 | 0 | NA | 4 | 168,333 | 2.83 (1.72, 3.94) |
| Low physical activity (%) | 0 | 0 | NA | 1 | 39,127 | -6.04 (-8.46, -3.63) |
| Vocational/high school (%) | 0 | 0 | NA | 2 | 94,649 | 2.2 (1.3, 3.1) |
| College/university (%) | 2 | 50,918 | 2.23 (2, 2.47) | 5 | 215,493 | 3.98 (-1.94, 9.89) |
| Alcohol (g/d, mean/median) | 2 | 60,680 | -7.32 (-18.34, 3.7) | 6 | 198,399 | -1.46 (-2.82, -0.09) |
| Red meat (g/d, mean/median) | 1 | 37,563 | -1.84 (-3, -0.68) | 5 | 1,945,47 | -2.82 (-4.85, -0.79) |
