# Supplementary material for: Health risk factors associated with meat, fruit and vegetable consumption in cohort studies: A comprehensive meta-analysis
Source: PLoS One. 2017 Aug 29;12(8):e0183787. doi: 10.1371/journal.pone.0183787 (PMC5574618; doi:10.1371/journal.pone.0183787)
Supplement: S16 Table — (DOCX) [file pone.0183787.s016.docx]

**Supplementary Table 16.** Summary associations between selected variables and vegetable consumption.

| Variables | No. of studies | No. of datasets | No. of cohorts | No. of individuals | Intercept (95% CI) | Slope per 100 g/d (95% CI) |
| --- | --- | --- | --- | --- | --- | --- |
| BMI (mean/median) | 18 | 24 | 19 | 917,885 | 24.31 (23.52, 25.1) | 0.03 (-0.18, 0.25) |
| BMI >30 (%) | 3 | 3 | 3 | 701,959 | 15.29 (9.8, 20.78) | 1.11 (0.22, 1.99) |
| BMI >25 (%) | 4 | 4 | 4 | 745,434 | 37 (22.06, 51.94) | 1.34 (0.48, 2.19) |
| Current smokers (%) | 15 | 22 | 17 | 1,406,513 | 27.25 (18.83, 35.67) | -4.01 (-5.9, -2.11) |
| Former smokers (%) | 8 | 12 | 9 | 907,782 | 26.17 (17.28, 35.05) | -0.07 (-4.78, 4.63) |
| Ever smokers (%) | 12 | 15 | 13 | 1,039,398 | 56.68 (45.15, 68.22) | -4.14 (-8.32, 0.03) |
| Never smokers (%) | 12 | 15 | 13 | 1,039,398 | 40.59 (30.14, 51.04) | 1.75 (0.43, 3.06) |
| High physical activity (%) | 7 | 10 | 8 | 928,377 | 24.03 (13.56, 34.49) | 6.07 (2.56, 9.58) |
| Low physical activity (%) | 3 | 3 | 3 | 676,895 | 28.66 (6.23, 51.1) | -0.71 (-3.51, 2.1) |
| Vocational/high school (%) | 3 | 3 | 3 | 589,530 | 27.6 (17.76, 37.44) | -1.34 (-6.01, 3.33) |
| College/university (%) | 9 | 11 | 9 | 1,002,204 | 21.46 (13.05, 29.88) | 3.72 (1.76, 5.68) |
| Alcohol (g/d, mean/median) | 7 | 8 | 7 | 288,146 | 15.78 (2.67, 28.88) | -1.23 (-2.39, -0.08) |
| Red meat (g/d, mean/median) | 7 | 8 | 7 | 433,288 | 53.63 (35.8, 71.45) | 0.41 (-0.9, 1.73) |
| Processed meat (g/d, mean/median) | 1 | 1 | 1 | 183,522 | 27.6 (25.86, 29.35) | -2.13 (-2.59, -1.67) |
