# Supplementary material for: Health risk factors associated with meat, fruit and vegetable consumption in cohort studies: A comprehensive meta-analysis
Source: PLoS One. 2017 Aug 29;12(8):e0183787. doi: 10.1371/journal.pone.0183787 (PMC5574618; doi:10.1371/journal.pone.0183787)
Supplement: S17 Table — NA, not applicable. (DOCX) [file pone.0183787.s017.docx]

**Supplementary Table 17.** Summary associations between selected variables and vegetable consumption, by sexes. NA, not applicable.

|  | Men |  |  | Women |  |  |
| --- | --- | --- | --- | --- | --- | --- |
| Variables | No. of cohorts | No. of individuals | Slope per 100 g/d (95% CI) | No. of cohorts | No. of individuals | Slope per 100 g/d (95% CI) |
| BMI (mean/median) | 7 | 146,439 | -0.11 (-0.29, 0.07) | 12 | 442,594 | -0.08 (-0.24, 0.07) |
| BMI >30 (%) | 0 | 0 | NA | 1 | 64,191 | 1.23 (0.97, 1.5) |
| BMI >25 (%) | 1 | 43,475 | 1.27 (1.05, 1.49) | 1 | 64,191 | 2.25 (1.89, 2.62) |
| Current smokers (%) | 7 | 122,322 | -6.66 (-11.42, -1.9) | 10 | 364,876 | -1.77 (-2.8, -0.75) |
| Former smokers (%) | 5 | 98,385 | 3.83 (-0.4, 8.06) | 4 | 100,536 | 1.07 (-1.1, 3.24) |
| Ever smokers (%) | 4 | 85,030 | -4.08 (-6.65, -1.51) | 6 | 207,887 | -0.85 (-3.59, 1.89) |
| Never smokers (%) | 4 | 85,030 | 3.65 (1.43, 5.87) | 6 | 207,887 | 0.75 (-1.81, 3.31) |
| High physical activity (%) | 2 | 44,773 | 8.31 (-1.42, 18.04) | 5 | 186,351 | 5.99 (1.72, 10.26) |
| Low physical activity (%) | 0 | 0 | NA | 1 | 39,127 | -3.5 (-4.58, -2.42) |
| Vocational/high school (%) | 0 | 0 | NA | 1 | 64,191 | 2.85 (1.47, 4.22) |
| College/university (%) | 2 | 50,918 | 3.32 (-3.01, 9.65) | 5 | 215,493 | 3.79 (0.53, 7.06) |
| Alcohol (g/d, mean/median) | 2 | 60,680 | -2.64 (-6.34, 1.06) | 5 | 167,981 | -0.4 (-1.15, 0.35) |
| Red meat (g/d, mean/median) | 1 | 37,563 | 2.48 (-3.9, 8.86) | 5 | 194,587 | 0.51 (-1.32, 2.35) |
