# Supplementary material for: Health risk factors associated with meat, fruit and vegetable consumption in cohort studies: A comprehensive meta-analysis
Source: PLoS One. 2017 Aug 29;12(8):e0183787. doi: 10.1371/journal.pone.0183787 (PMC5574618; doi:10.1371/journal.pone.0183787)
Supplement: S18 Table — NA, not applicable. (DOCX) [file pone.0183787.s018.docx]

**Supplementary Table 18.** Summary associations between selected variables and fruit consumption, by geographical region. NA, not applicable.

|  | Europe |  |  | US |  |  | Asia |  |  |
| --- | --- | --- | --- | --- | --- | --- | --- | --- | --- |
| Variables | No. of cohorts | No. of individuals | Slope per 100 g/d (95% CI) | No. of cohorts | No. of individuals | Slope per 100 g/d (95% CI) | No. of cohorts | No. of individuals | Slope per 100 g/d (95% CI) |
| BMI (mean/median) | 4 | 161,723 | -0.1 (-0.15, -0.05) | 8 | 365,967 | -0.24 (-0.56, 0.09) | 4 | 191,295 | 0.69 (-0.35, 1.73) |
| BMI >30 (%) | 1 | 486,799 | 1.8 (1.14, 2.46) | 0 | 0 | NA | 1 | 64,191 | 0.31 (-0.55, 1.17) |
| BMI >25 (%) | 1 | 486,799 | 2.41 (1.69, 3.13) | 0 | 0 | NA | 1 | 64,191 | 1.4 (-0.02, 2.81) |
| Current smokers (%) | 3 | 517,987 | -8.93 (-22, 4.15) | 6 | 293,709 | -3.53 (-5.01, -2.05) | 4 | 191,295 | -1.43 (-9.22, 6.36) |
| Former smokers (%) | 1 | 486,799 | 0 (-0.7, 0.69) | 2 | 119,020 | -0.36 (-0.7, -0.02) | 2 | 67,619 | -1.91 (-8.05, 4.24) |
| Ever smokers (%) | 3 | 617,334 | -2.8 (-4.39, -1.21) | 6 | 232,522 | -6.07 (-10.8, -1.34) | 1 | 38,540 | 5.18 (-10.35, 20.71) |
| Never smokers (%) | 3 | 617,334 | 2.26 (0.13, 4.39) | 6 | 232,522 | 6.07 (1.35, 10.8) | 1 | 38,540 | 42.79 (35.81, 49.76) |
| High physical activity (%) | 3 | 600,513 | 2.87 (-0.01, 5.76) | 2 | 73,684 | 3.55 (2.05, 5.05) | 2 | 123,676 | 6.93 (-3.6, 17.46) |
| Low physical activity (%) | 1 | 486,799 | 0.82 (0.51, 1.14) | 1 | 39,127 | -6.04 (-8.46, -3.63) | 0 | 0 | NA |
| Vocational/high school (%) | 2 | 517,257 | 0.24 (-2.71, 3.19) | 0 | 0 | NA | 2 | 102,731 | 17.13 (-11.24, 45.5) |
| College/university (%) | 1 | 486,799 | 0 (-1.18, 1.18) | 3 | 173,141 | 5.18 (-1.93, 12.3) | 4 | 191,295 | 8.83 (-2.88, 20.53) |
| Alcohol (g/d, mean/median) | 1 | 30,458 | -0.3 (-0.4, -0.2) | 5 | 199,542 | -1.46 (-3, 0.09) | 2 | 88,564 | -7.3 (-13.6, -1.01) |
| Red meat (g/d, mean/median) | 0 | 0 | NA | 6 | 249,731 | -3.1 (-4.77, -1.43) | 0 | 0 | NA |
