# Supplementary material for: Health risk factors associated with meat, fruit and vegetable consumption in cohort studies: A comprehensive meta-analysis
Source: PLoS One. 2017 Aug 29;12(8):e0183787. doi: 10.1371/journal.pone.0183787 (PMC5574618; doi:10.1371/journal.pone.0183787)
Supplement: S19 Table — (DOCX) [file pone.0183787.s019.docx]

**Supplementary Table 19.** Summary associations between selected variables and vegetable consumption, by geographical region.

|  | Europe |  |  | US |  |  | Asia |  |  |
| --- | --- | --- | --- | --- | --- | --- | --- | --- | --- |
| Variables | No. of cohorts | No. of individuals | Slope per 100 g/d (95% CI) | No. of cohorts | No. of individuals | Slope per 100 g/d (95% CI) | No. of cohorts | No. of individuals | Slope per 100 g/d (95% CI) |
| BMI (mean/median) | 3 | 77,706 | 0 (-0.64, 0.64) | 9 | 549,496 | -0.08 (-0.25, 0.09) | 7 | 290,683 | 0.15 (-0.28, 0.58) |
| BMI >30 (%) | 1 | 486,799 | 1.82 (0.5, 3.13) | 0 | 0 | NA | 1 | 64,191 | 1.23 (0.97, 1.5) |
| BMI >25 (%) | 1 | 486,799 | 1.65 (0.01, 3.29) | 0 | 0 | NA | 2 | 107,666 | 1.76 (0.79, 2.73) |
| Current smokers (%) | 2 | 487,528 | -4.67 (-8.2, -1.13) | 7 | 477,333 | -1.6 (-2.15, -1.04) | 7 | 290,683 | -5.95 (-9.12, -2.78) |
| Former smokers (%) | 1 | 486,799 | -0.03 (-0.9, 0.84) | 3 | 302,542 | 1.29 (-0.71, 3.28) | 5 | 118,441 | -0.87 (-9.1, 7.36) |
| Ever smokers (%) | 2 | 534,087 | -2.43 (-3.35, -1.51) | 7 | 415,949 | -0.44 (-1.73, 0.84) | 4 | 89,362 | -10.91 (-21.89, 0.07) |
| Never smokers (%) | 2 | 534,087 | 1.52 (0.69, 2.36) | 7 | 415,949 | 0.44 (-0.84, 1.73) | 4 | 89,362 | 4.04 (1.68, 6.4) |
| High physical activity (%) | 1 | 486,799 | 0.57 (-0.12, 1.26) | 2 | 73,594 | 4.07 (-0.53, 8.67) | 4 | 217,015 | 8.73 (4.31, 13.15) |
| Low physical activity (%) | 1 | 486,799 | 1.21 (0.78, 1.64) | 1 | 39,127 | -3.5 (-4.58, -2.42) | 0 | 0 | NA |
| Vocational/high school (%) | 1 | 486,799 | -1.71 (-1.94, -1.49) | 0 | 0 | NA | 2 | 102,731 | -1.16 (-9.01, 6.69) |
| College/university (%) | 1 | 486,799 | 2.23 (0.2, 4.27) | 3 | 173,141 | 6.09 (3.64, 8.55) | 4 | 191,295 | 2.89 (-0.22, 6.01) |
| Alcohol (g/d, mean/median) | 0 | 0 | NA | 5 | 199,582 | -0.34 (-1.06, 0.37) | 2 | 88,564 | -2.72 (-4.75, -0.7) |
| Red meat (g/d, mean/median) | 0 | 0 | NA | 7 | 433,288 | 0.41 (-0.9, 1.73) | 0 | 0 | NA |
| Processed meat (g/d, mean/median) | 0 | 0 | NA | 1 | 183,522 | -2.13 (-2.59, -1.67) | 0 | 0 | NA |
