# Supplementary figures and images for: Health risk factors associated with meat, fruit and vegetable consumption in cohort studies: A comprehensive meta-analysis
Source: PLoS One. 2017 Aug 29;12(8):e0183787. doi: 10.1371/journal.pone.0183787 (PMC5574618; doi:10.1371/journal.pone.0183787)

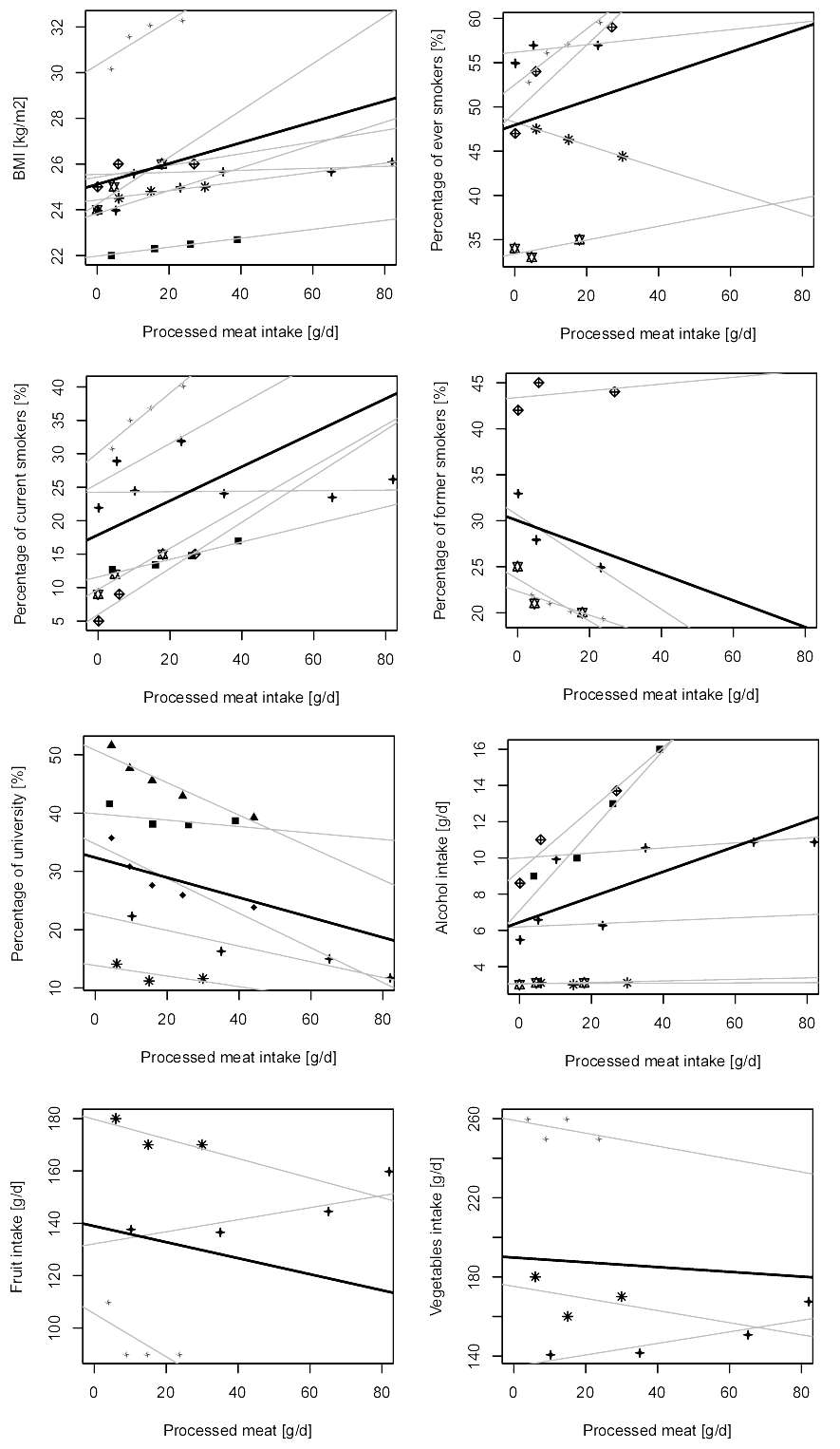

Supplement: S1 Fig — Symbols represent different cohorts; light lines represent linear regression coefficients of individual studies; bold lines represent summary estimates average increase of each variable for increase of red meat intake. (TIF) [file pone.0183787.s020.tif]

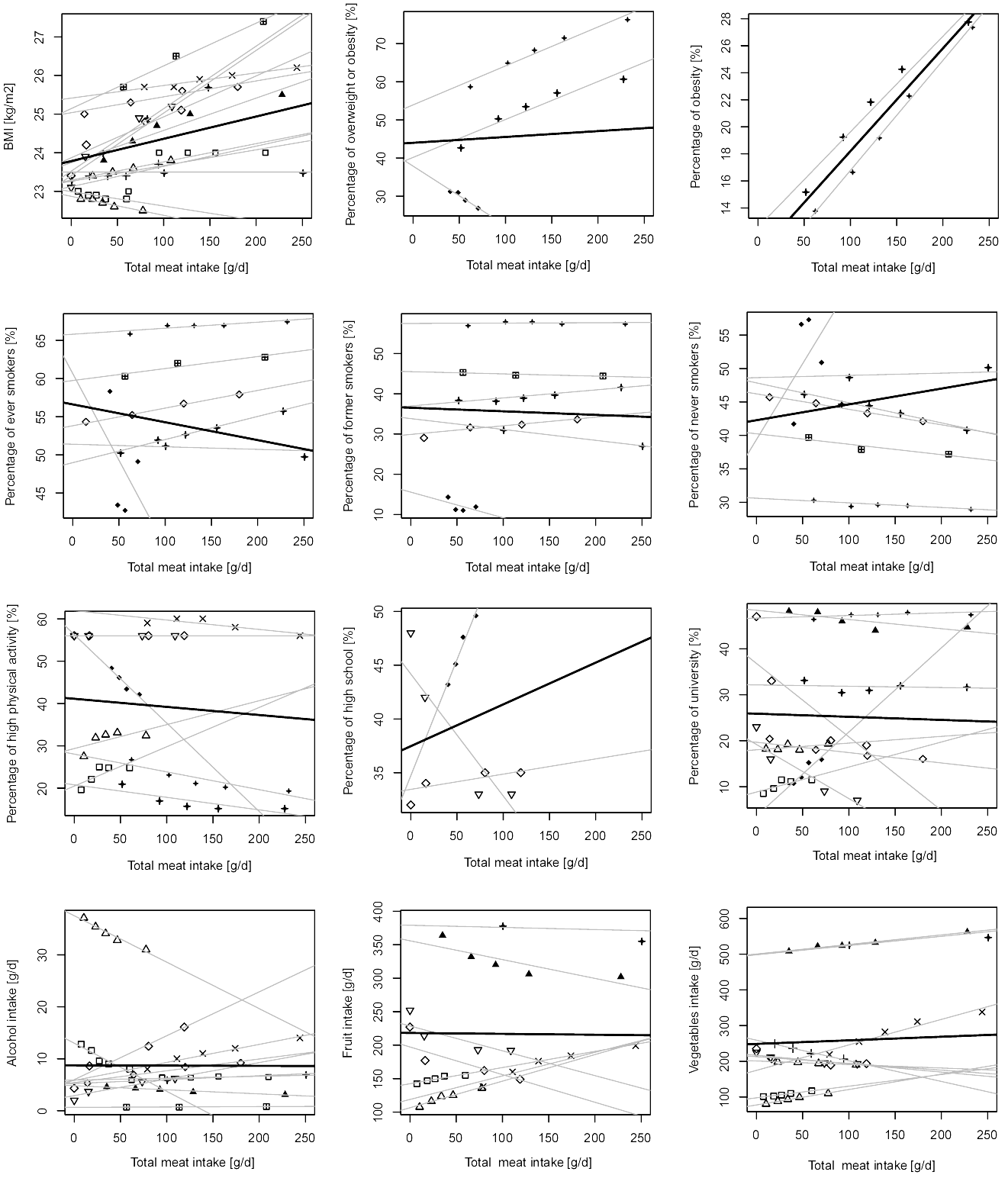

Supplement: S2 Fig — Symbols represent different cohorts; light lines represent linear regression coefficients of individual studies; bold lines represent summary estimates average increase of each variable for increase of red meat intake. (TIF) [file pone.0183787.s021.tif]

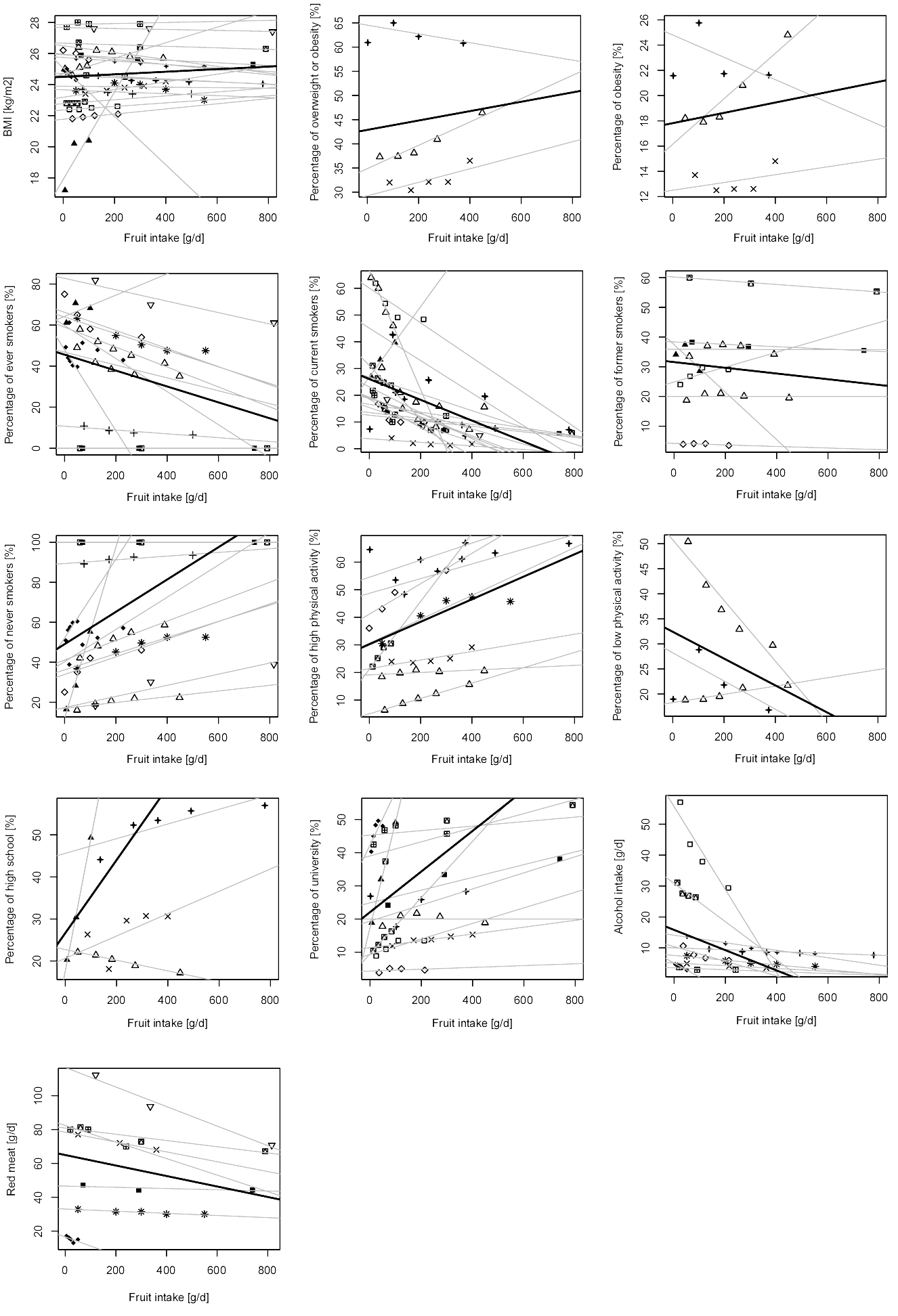

Supplement: S3 Fig — Symbols represent different cohorts; light lines represent linear regression coefficients of individual studies; bold lines represent summary estimates average increase of each variable for increase of fruit and vegetable intake. (TIF) [file pone.0183787.s022.tif]

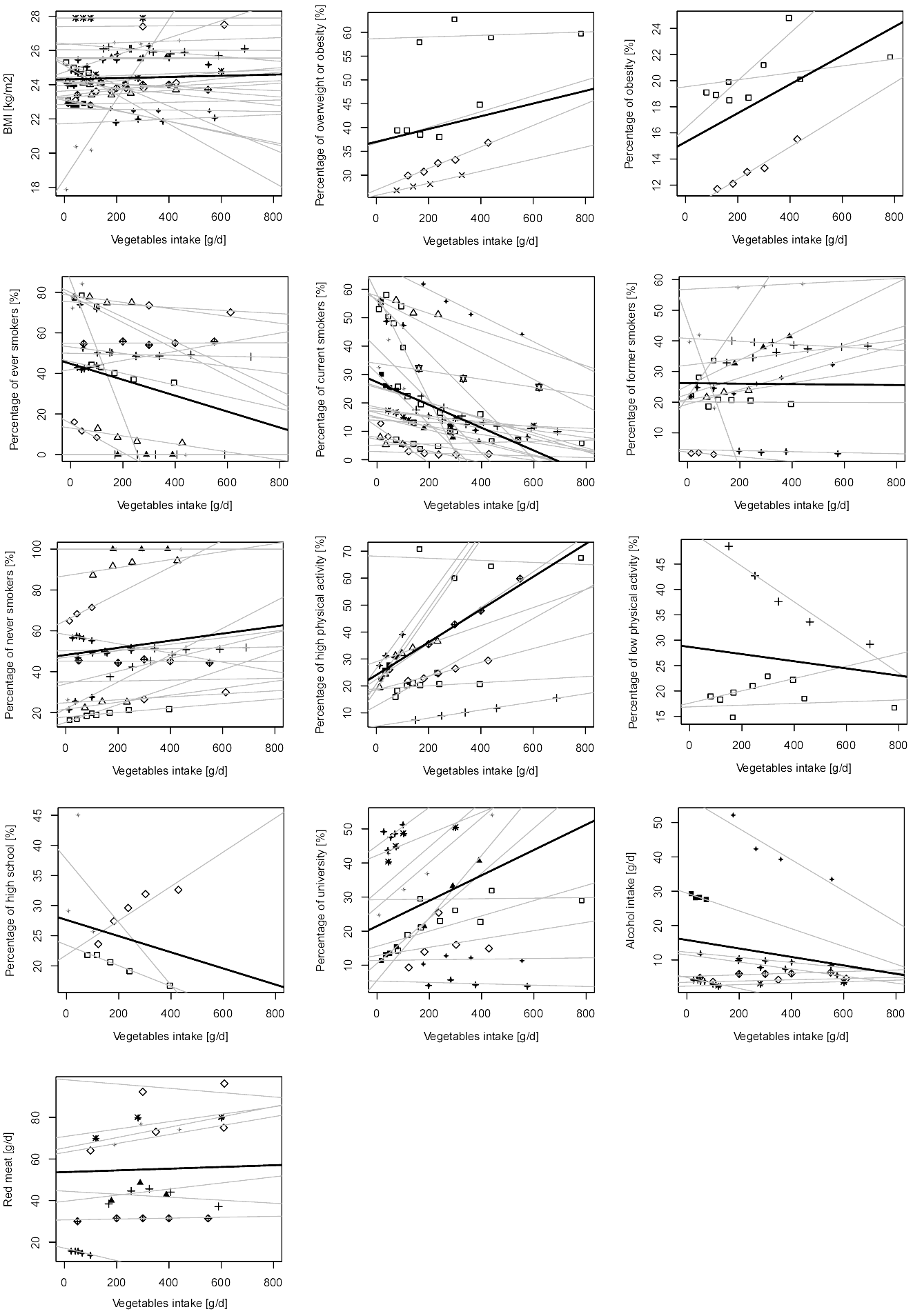

Supplement: S4 Fig — Symbols represent different cohorts; light lines represent linear regression coefficients of individual studies; bold lines represent summary estimates average increase of each variable for increase of fruit and vegetable intake. (TIF) [file pone.0183787.s023.tif]
